# Comparative genomics of bdelloid rotifers: Insights from desiccating and nondesiccating species

**DOI:** 10.1371/journal.pbio.2004830

**Published:** 2018-04-24

**Authors:** Reuben W. Nowell, Pedro Almeida, Christopher G. Wilson, Thomas P. Smith, Diego Fontaneto, Alastair Crisp, Gos Micklem, Alan Tunnacliffe, Chiara Boschetti, Timothy G. Barraclough

**Affiliations:** 1 Department of Life Sciences, Imperial College London, Silwood Park Campus, Ascot, Berkshire, United Kingdom; 2 National Research Council of Italy, Institute of Ecosystem Study, Verbania Pallanza, Italy; 3 Department of Chemical Engineering and Biotechnology, West Cambridge Site, University of Cambridge, Cambridge, United Kingdom; 4 Department of Genetics, Cambridge Systems Biology Centre, Downing Site, University of Cambridge, Cambridge, United Kingdom; 5 School of Biological and Marine Sciences, Plymouth University, Portland Square Building, Plymouth, United Kingdom; The Wellcome Trust Sanger Institute, United Kingdom of Great Britain and Northern Ireland

## Abstract

Bdelloid rotifers are a class of microscopic invertebrates that have existed for millions of years apparently without sex or meiosis. They inhabit a variety of temporary and permanent freshwater habitats globally, and many species are remarkably tolerant of desiccation. Bdelloids offer an opportunity to better understand the evolution of sex and recombination, but previous work has emphasised desiccation as the cause of several unusual genomic features in this group. Here, we present high-quality whole-genome sequences of 3 bdelloid species: *Rotaria macrura* and *R*. *magnacalcarata*, which are both desiccation intolerant, and *Adineta ricciae*, which is desiccation tolerant. In combination with the published assembly of *A*. *vaga*, which is also desiccation tolerant, we apply a comparative genomics approach to evaluate the potential effects of desiccation tolerance and asexuality on genome evolution in bdelloids. We find that ancestral tetraploidy is conserved among all 4 bdelloid species, but homologous divergence in obligately aquatic *Rotaria* genomes is unexpectedly low. This finding is contrary to current models regarding the role of desiccation in shaping bdelloid genomes. In addition, we find that homologous regions in *A*. *ricciae* are largely collinear and do not form palindromic repeats as observed in the published *A*. *vaga* assembly. Consequently, several features interpreted as genomic evidence for long-term ameiotic evolution are not general to all bdelloid species, even within the same genus. Finally, we substantiate previous findings of high levels of horizontally transferred nonmetazoan genes in both desiccating and nondesiccating bdelloid species and show that this unusual feature is not shared by other animal phyla, even those with desiccation-tolerant representatives. These comparisons call into question the proposed role of desiccation in mediating horizontal genetic transfer.

## Author summary

Bdelloid rotifers are microscopic animals that live in freshwater habitats throughout the world. Two life history characteristics distinguish these common invertebrates as extraordinary. First, they have existed for millions of years apparently without sex: males have not been reported, and females produce genetically identical daughters via parthenogenesis. Second, most bdelloid species are highly resilient to desiccation and can survive without water for extended periods of time (a process known as anhydrobiosis). These 2 attributes have been predicted to leave signatures in bdelloid genomes. Here, we present new draft genomes for 3 bdelloid species and employ comparative genomics to explore the potential impacts of bdelloids’ unusual lifestyle on genome structure and content. We find that many proposed genomic consequences of asexuality and desiccation tolerance do not hold true for all species. The genomes of bdelloids may be more similar to those of other animals than previously thought, though a remarkable exception is the high proportion of genes acquired horizontally from nonmetazoan taxa. Our findings necessitate a reevaluation of the effects of asexuality and desiccation on genome evolution in bdelloid rotifers.

## Introduction

The bdelloid rotifers are a class of microscopic invertebrates found in freshwater habitats worldwide. Two life history characteristics make these soft-bodied filter feeders unusual among animals. First, bdelloids famously lack males [1] or cytological evidence of meiosis [2,3] and are only known to reproduce via mitotic parthenogenesis. They are therefore one of the best-substantiated examples of a eukaryotic taxon that has evolved apparently without sex or meiosis for tens of millions of years [1,2,4,5]. Famously labelled ‘an evolutionary scandal’ [6], bdelloids have diversified into over 500 species [7,8] defying the usual fate of asexual lineages [9–11]. Their persistence has implications for theories of the evolution of sex and recombination, a fundamental puzzle in biology [12–15]. A second key feature is that most bdelloid species are remarkably tolerant of desiccation and can survive the loss of almost all cellular water at any stage in their life cycle, including as adults [16,17]. As water evaporates, animals contract their bodies into flat, ellipsoid ‘tuns’ and enter a dormant state called anhydrobiosis, during which all metabolic activities associated with life are suspended [5,16,18]. Individuals can remain in this condition for long periods, usually days or weeks but occasionally several years [19,20]. The return of water restores metabolism and reproduction, with little evidence of negative fitness consequences for survivors [21]. Species that live in limnoterrestrial habitats such as puddles, leaf litter, and moss are subject to rapid and repeated cycles of drying. The ability to survive desiccation has been proposed to play a key role in bdelloid evolution [5,22].

Early marker-based analyses of bdelloid genomes recovered highly divergent gene copies that were interpreted as nonrecombining descendants of ancient former alleles [4]. Along with the low copy number of vertically inherited transposable elements (TEs) [23], this result was considered positive genetic evidence of long-term asexual evolution. However, subsequent investigations of larger genomic regions revealed evidence of tetraploidy, probably arising from an ancient hybridisation or genome duplication event affecting diploid ancestors prior to the diversification of bdelloid families [5,24,25]. Genes generally have up to 4 copies, arranged as 2 pairs, with greater divergence between pairs (‘ohnologs’, also known as homeologs in other polyploid systems) than within pairs (‘homologs’) [5,24,25]. Another extraordinary feature was that a remarkably high proportion of bdelloid genes show similarity to nonmetazoan orthologs, mostly from bacteria but also fungi and plants, suggesting a rate of horizontal gene transfer (HGT) into bdelloid genomes at least an order of magnitude greater than that observed in other eukaryotes [26]. Many genes originating by HGT from nonmetazoans are expressed and functional [26,27].

The first whole-genome sequence for a bdelloid substantiated many of these findings [28]. The tetraploid genome of *Adineta vaga* comprises both homologous regions with low divergence (median 1.4% for protein-coding genes) and conserved gene order (i.e., high collinearity) as well as ohnologous regions with much higher divergence (median 24.9%) and degeneration of gene order (i.e., low collinearity). The genome encodes remarkably few TEs (approximately 3% of the genome) but a high proportion of foreign genes (approximately 8%), many of which occur as quartets and were therefore presumably acquired prior to tetraploidisation. Unusual structural features were also reported, including a large number of breaks in collinearity between homologous regions and linkage of homologs on the same assembly scaffold, often arranged as genomic palindromes. This assembly cannot, therefore, be decomposed into haploid sets, a finding that was interpreted as further evidence of long-term ameiotic evolution in *A*. *vaga* [28].

To what extent are the genomic characteristics of bdelloid rotifers explained by their unusual biology and ecology? An important discovery was that bdelloids can survive doses of ionising radiation that would be lethal to nearly any other animal, owing to their ability to repair the resulting DNA double-strand breaks (DSBs) and recover from extensive genome fragmentation [29,30]. Experiments in *A*. *vaga* showed that comparable genome fragmentation also occurs during desiccation, and this led to the view that bdelloid genomes may be shaped by the need for recovering animals to repair DSBs arising from repeated desiccation [31]. One hypothesis is that homologous gene copies are used as reciprocal templates for repairing DSBs, a process that would act to homogenise homologous regions periodically via gene conversion and select against individuals with excessive divergence because template mismatches would disrupt DNA repair [24,28–31]. In this scenario, the molecular consequences of desiccation are directly linked to the patterns of intragenomic divergence observed in bdelloid genomes, via breakage and repair of DNA. However, the link between desiccation and DSBs is not unequivocal, and evidence from other anhydrobiotic taxa is mixed. For example, DNA integrity is largely maintained in desiccating tardigrades [32–34], but not in the chironomid midge *Polypedilum vanderplanki* [35]. In bdelloids, a key prediction is that species that undergo desiccation more frequently should experience higher rates of DSB repair, resulting in more opportunities for gene conversion and thus a lower level of homologous divergence.

A related hypothesis is that foreign DNA present in the environment may become incorporated into bdelloid genomes via nonhomologous recombination during DSB repair following desiccation, resulting in a higher rate of HGT than is experienced by other eukaryotes [26,28,30,31]. Evidence for high levels of nonhomologous transfer inspired further suggestions that DSB repair might even facilitate homologous horizontal transfer and genetic exchange between individual animals [26,28,30,31]. These ideas remain controversial, however, and recent claims of evidence for DNA transfer between individuals of *A*. *vaga* [36] have subsequently been identified as artefacts of experimental cross-contamination [37]. A separate recent study reported a striking pattern of allele sharing among 3 individuals of another bdelloid species, *Macrotrachela quadricornifera*, which was interpreted as evidence of sexual reproduction via an unusual form of meiosis (similar to that of plants in the genus *Oenothera*) [38,39]. However, no evidence for such a mechanism was apparent in a larger study of the genus *Adineta* [40]. In the absence of clear evidence for either occasional sex or horizontal genetic transfer between individual bdelloids (but without discounting either possibility), the nature of bdelloid recombination remains an open question.

**Fig 1:**
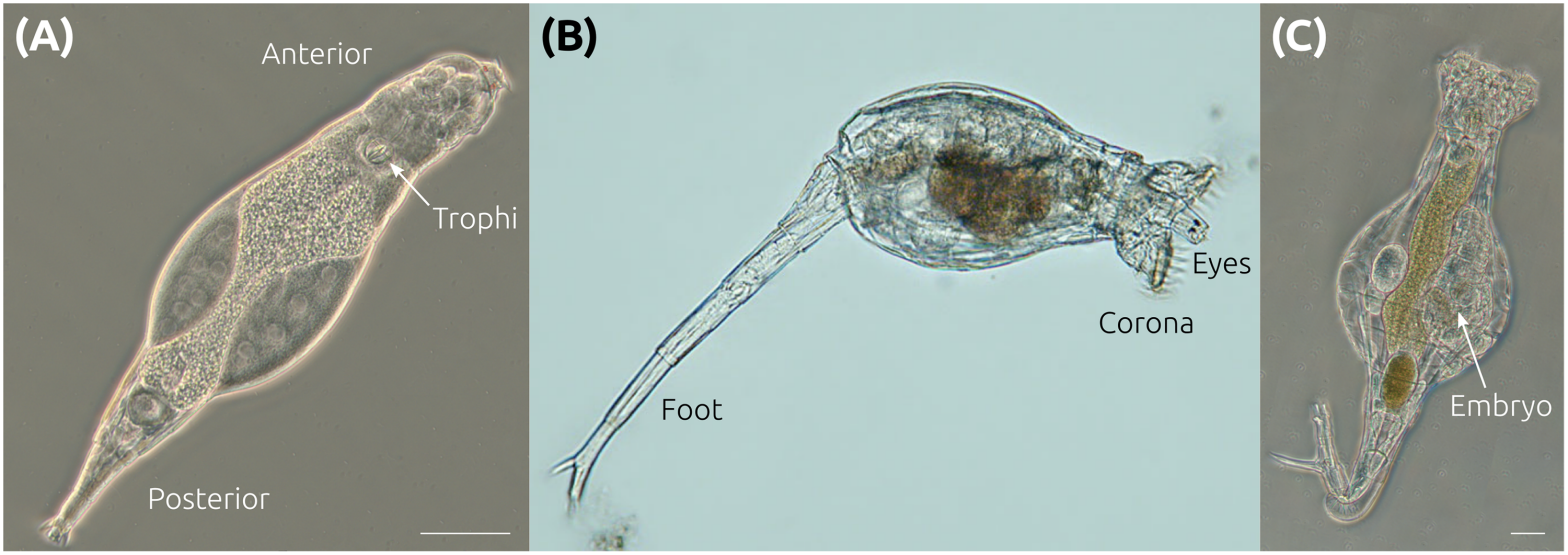
Bdelloid rotifer morphology. (A) *A*. *ricciae* individual in phase contrast, showing anterior–posterior axis and trophi (jaws). This genus does not possess the ciliated wheel-like organs (corona) on the head that distinguish other rotifers. (B) *R*. *macrura* showing eyes, extended corona used for feeding and locomotion, and a foot used for substrate attachment. (C) *R*. *magnacalcarata* with embryo developing internally. Scale bars indicate 50 μm; note: *R*. *macrura* is approximately the same size as *R*. *magnacalcarata*. Panels A and C courtesy of C. G. Wilson; panel B courtesy of M. Plewka (www.plingfactory.de).

Showing both extensive anhydrobiotic capabilities and putatively ancient asexuality, bdelloid rotifers sit at a unique junction in animal evolution. To better understand the relative contributions of these features to bdelloid genome evolution, we have taken advantage of natural variation in the capacity of species to survive desiccation by sampling and comparing whole genomes from multiple taxa. In particular, many species in the genus *Rotaria* live in permanent water bodies and do not survive desiccation in the laboratory [17,41]. Here, we present high-coverage, high-quality genome sequences for 3 species from 2 genera: the desiccation-tolerant species *A*. *ricciae* and the obligately aquatic, nondesiccating species *R*. *macrura* and *R*. *magnacalcarata* (Fig 1). These are compared with the published genome of *A*. *vaga*. Using a range of assembly approaches, we confirm the conservation of ancestral tetraploidy in all species but demonstrate substantial variation in genome size among species. We then test predictions regarding the effects of desiccation tolerance on intragenomic homologous divergence and investigate genome architecture within species to ask whether the unusual genomic structures observed in *A*. *vaga* are a general feature across bdelloids. Finally, we contrast a range of genome characteristics, including homologous divergence, HGT content, and repeat abundance across a wider range of animal taxa, allowing us to place some of the unique features of bdelloid genomes in a wider metazoan context.

## Results and discussion

### Reference genome assembly and annotation

Reference genome sequences for *A*. *ricciae*, *R*. *macrura*, and *R*. *magnacalcarata* were assembled using a combination of long- and short-read sequencing technologies (S1 Table). Kmer spectra of raw and filtered sequencing reads indicated high (>100x) but variable coverage across sites in each genome (S1 Fig). In addition, a large proportion of low-coverage kmers indicated substantial polymorphism in the *R*. *magnacalcarata* raw data, most likely corresponding to population variation in the multi-individual DNA sample collected for this species (see Materials and methods). Contaminating reads from non–target organisms were excluded by scrutinising initial draft assemblies for contigs showing abnormal guanine-cytosine (GC) content, coverage, or taxonomic annotations, removing 3.1% of reads from the *R*. *macrura* dataset, 2.1% from *R*. *magnacalcarata*, and 6.3% from *A*. *ricciae* (including approximately 9 Mb sequences annotated as *Pseudomonas* spp.) (S2 Fig). Given the complex patterns of intragenomic divergence and gene copy number observed in other bdelloid species [24,25,28], we adopted 2 assembly strategies. First, reference assemblies were generated with a focus on high assembly contiguity. Reference sequences were constructed using the Platanus assembler [42] and improved using Redundans [43] (S3 and S4 Figs). Second, ‘maximum haplotype’ assemblies were generated with a focus on maximum separation and resolution of homologous regions, even if this reduced assembly contiguity. This was intended to minimise the confounding effects of assembly ‘collapse’, a phenomenon whereby homologous regions with no or low divergence are assembled as a single contig with 2-fold coverage relative to separately assembled regions. Further assembly metrics are provided in S1 Data. All assemblies have been submitted to DDBJ/ENA/GenBank under the project accession ID PRJEB23547.

**Fig 2:**
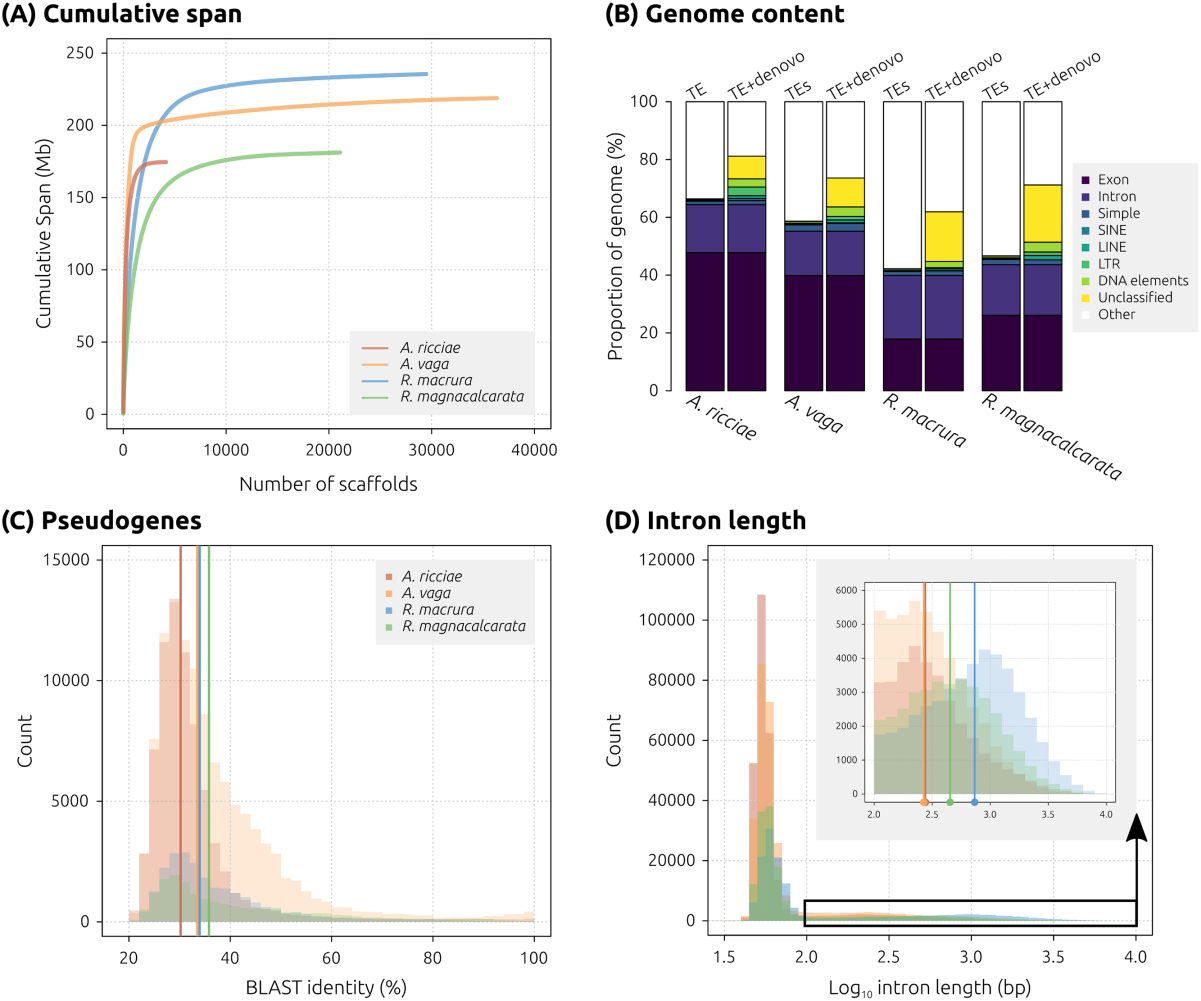
Genome properties of bdelloid rotifers. (A) Assembly contiguity. Cumulative assembly span for *A*. *ricciae* (red), *R*. *macrura* (blue), and *R*. *magnacalcarata* (green) reference assemblies, compared to the published sequence of *A*. *vaga* (orange). Scaffolds are arranged in descending length order along the x-axis, with cumulative span plotted along the y-axis. More contiguous assemblies achieve their total span with a smaller number of scaffolds, represented by a steeper line with a smaller tail. (B) Genome content. Proportion of each reference assembly covered by exons, introns, and identified repeat elements based on known metazoan TEs only (left-hand column) and TEs plus unclassified repeats detected using RepeatModeler (right-hand column). This shows that the inclusion of ab initio repeats results in substantially greater repeat content in all species, particularly in *Rotaria*. (C) Pseudogene detection. Distribution of percent identity for TBLASTN alignments (*E*-value ≤1 × 10^−20^) of predicted proteins to their own genome, discounting hits that overlapped with any existing predicted gene model. Only hits with a query coverage ≥95% are plotted. Vertical coloured bars indicate median values. (D) Intron length distributions for predicted genes (species coloured as previously). The inset shows detail of the upper tail of the main distribution (black box, truncated at ≥2.0). Vertical bars indicate median values for each species (of truncated distributions). Note log_10_ scale on y-axis. LINE, long interspersed element; LTR, long terminal repeat; SINE, short interspersed element; TE, transposable element.

Genome metrics for *A*. *ricciae*, *R*. *macrura*, and *R*. *magnacalcarata* reference assemblies are shown in Table 1, alongside those for the published assembly of *A*. *vaga* (accession GCA_000513175.1, hereafter referred to as the ‘2013’ assembly) [28]. The reference assembly of *A*. *ricciae* spanned 174.5 Mb, comprising 4,125 scaffolds with an N50 (length-weighted median) scaffold length of 276.8 kb (Fig 2A). The reference assemblies for *R*. *macrura* and *R*. *magnacalcarata* spanned 234.7 and 180.5 Mb over 29,255 and 20,900 scaffolds, respectively, with N50 scaffold lengths of 73.2 and 53.3 kb. The proportion of undetermined bases (i.e., gaps denoted as Ns) was low in all cases, accounting for 2.1%, 0.3%, and 0.8% of the *A*. *ricciae*, *R*. *macrura*, and *R*. *magnacalcarata* reference assemblies, respectively. The GC content was 35.6% for *A*. *ricciae*, 32.6% for *R*. *macrura*, and 31.9% for *R*. *magnacalcarata*; therefore, GC content in *A*. *ricciae* is higher relative to *A*. *vaga* (30.8%) and both *Rotaria* species. Gene completeness was assessed by comparing sets of core eukaryotic genes to each reference assembly using Core Eukaryotic Gene Mapping Approach (CEGMA) and Benchmarking Universal Single-Copy Orthologs (BUSCO) [44,45]. Recovery of full-length CEGMA genes (*n* = 248) was 98%, 94%, and 98% for *A*. *ricciae*, *R*. *macrura*, and *R*. *magnacalcarata*, respectively, and gene duplication (average copy number per CEGMA gene) was 2.9, 1.6, and 1.7, respectively. The equivalent recovery of a larger set of BUSCO core metazoan genes (*n* = 978) was 90% for all assemblies, with duplication scores of 2.0, 1.2, and 1.2, respectively. The equivalent completeness and duplication scores for the *A*. *vaga* 2013 assembly were 96% and 3.0 for CEGMA, and 91% and 2.0 for BUSCO.

**Table 1:** Genome assembly and annotation metrics.

**Assembly**				
**Species**	***A*. *ricciae***	***A*. *vaga***^a^	***R*. *macrura***	***R*. *magnacalcarata***
**Accession (assembly name)**	GCA_900240375.1 (nAr.v1.8)	GCA_000513175.1 (‘2013’)	GCA_900239685.1 (nRc.v1.3)	GCA_900239745.1 (nRg.v1.8)
**Coverage**^b^ **(mean)**	134.2	Not calculated	210.0	76.3
**Span (Mb)**	174.5	217.9	234.7	180.5
**No. contigs**	10,421	36,335	31,105	29,824
**Contig N50**^c^ **(kb)**	58.9	96.7	58.3	24.7
**No. scaffolds**	4,125	36,167	29,255	20,900
**Scaffold N50 (kb) (#N50**^d^**)**	276.8 (#176)	260.3 (#240)	73.2 (#828)	53.3 (#890)
**Scaffold N90 (kb) (#N90)**	34.5 (#868)	7.8 (#1,470)	7.4 (#4,527)	5.3 (#4,560)
**Scaffold longest (kb)**	1,675.3	1,087.3	692.9	531.4
**Gaps (Ns) span (kb) (% genome)**	3,668.1 (2.1%)	4,136.3 (1.9%)	640.4 (0.3%)	1,351.6 (0.8%)
**% GC**	35.6	30.8	32.6	31.9
**CEGMA**^e^ **(*n* = 248)**	C: 98%; F: 99%; D: 2.9	C: 96%; F: 97%; D: 3.0	C: 94%; F: 98%; D: 1.6	C: 98%; F: 99%; D: 1.7
**BUSCO**_**EUK**_ **(*n* = 303)**	C: 97%; F: 98%; D: 2.1	C: 97%; F: 98%; D: 2.0	C: 95%; F: 98%; D: 1.2	C: 96%; F: 97%; D: 1.2
**BUSCO**_**MET**_ **(*n* = 978)**	C: 90%; F: 92%; D: 2.0	C: 91%; F: 92%; D: 2.0	C: 90%; F: 91%; D: 1.2	C: 90%; F: 91%; D: 1.2
**Reads mapped (raw) (%)**	85.7	Not calculated	98.5	93.4
**Transcripts mapped**^f^ **(%)**	99.1	Not calculated	NA	98.4
**Annotation**				
**Species**	***A*. *ricciae***	***A*. *vaga***	***R*. *macrura***	***R*. *magnacalcarata***
**Method**	BRAKER	MAKER/Augustus	MAKER/Augustus	BRAKER
**No. genes**	55,801	67,364	26,284	36,377
**No. CDS (includes isoforms)**	58,489	67,364	26,284	37,380
**No. CDS hit to UniProt90 (Diamond *E*-value ≤1 × 10**^**−5**^**)**	44,974 (77%)	49,915 (74%)	21,014 (80%)	26,542 (71%)
**CDS mean length (median) (bp)**	1,426.0 (1,059)	1,290.4 (945)	1,596.5 (1,197)	1,260.7 (948)
**No. HQ CDS**^g^	49,857	57,431	24,594	29,359
**Intron mean frequency (introns/gene)**	4.8	4.1	5.3	4.0
**Intron mean length (median) (bp)**	104.1 (55)	118.2 (58)	362.3 (67)	208.4 (61)
**Gene density (per Mb)**	319.8	300.3	112.0	201.5
**Mean intergenic distance (median) (kb)**	1.2 (0.6)	1.4 (0.8)	3.9 (2.7)	2.2 (1.4)
**Exon span (Mb) (%)**	83.4 (47.8%)	86.9 (39.9%)	42.0 (17.9%)	47.1 (26.1%)
**Intron span (Mb) (%)**	29.0 (16.6%)	33.4 (15.3%)	51.6 (22.0%)	31.5 (17.5%)
**No. SEG**^h^ (%)	12,250 (20.9%)	16,504 (24.5%)	1,149 (4.3%)	9,481 (25.3%)
**Transcript % GC**	38.2	32.8	35.0	34.3
**BUSCO**_**EUK**_ **(*n* = 303)**	C: 96%; F: 99%; D: 2.4	C: 97%; F: 98%; D: 2.3	C: 96%; F: 98%; D: 1.2	C: 99%; F: 100%; D:1.2
**BUSCO**_**MET**_ **(*n* = 978)**	C: 92%; F: 94%; D: 2.4	C: 92%; F: 93%; D: 2.3	C: 91%; F: 93%; D: 1.3	C: 94%; F: 96%; D: 1.3

^a^Assembly metrics for *A*. *vaga* are based on assembly GCA_000513175.1 by Flot et al. (2013) [28].

^b^Average read coverage based on trimmed and filtered data.

^c^Length-weighted median scaffold length; i.e., the length of the scaffold/contig at which 50% of the total genome span is reached.

^d^The number of scaffolds required to reach N50.

^e^CEGMA/BUSCO notation: C, proportion of genes completely recovered; F, proportion of genes at least partially recovered (includes complete genes); D, duplication rate (average number of copies recovered); *n*, number of genes tested.

^f^Proportion of transcripts with a significant BLAT [46] alignment to the genome.

^g^After removal of short genes (<30 bases) with either no hit to UniRef90 or similarity to known TEs.

^h^Genes without introns.

Abbreviations: BLAT, BLAST-like alignment tool; BUSCO, Benchmarking Universal Single-Copy Orthologs; CDS, coding sequence; CEGMA, Core Eukaryotic Gene Mapping Approach; EUK, Eukaryota; GC, guanine-cytosine; HQ, high-quality; MET, Metazoa; SEG, single-exon gene; TE, transposable element.

The number of genes predicted from each reference assembly varied considerably among species. Gene prediction was performed using BRAKER [47] if RNA sequencing (RNASeq) data were available or MAKER/Augustus [48,49] if not, giving initial estimates of 55,801, 26,284, and 36,377 protein-coding genes for *A*. *ricciae*, *R*. *macrura*, and *R*. *magnacalcarata*, respectively (Table 1). Genes with BLAST matches to TEs (*E*-value ≤1 × 10^−5^) and short genes with no matches to UniProt90 (i.e., likely spurious gene models) were removed, resulting in ‘high-quality’ sets of 49,857, 24,594, and 29,359 protein-coding genes for downstream analyses (Table 1; S2 Data). Reannotation of the *A*. *vaga* 2013 assembly using MAKER/Augustus resulted in 67,364 predicted genes, reducing to 57,431 after quality control (S2 Data). Therefore, the reference genomes of *R*. *macrura* and *R*. *magnacalcarata* appear to encode approximately half the number of genes observed in *A*. *ricciae* and *A*. *vaga*. Correspondingly, the mean intergenic distance was higher in *R*. *macrura* (mean 3.9 kb) and *R*. *magnacalcarata* (2.2 kb) than in *A*. *vaga* (1.4 kb) or *A*. *ricciae* (1.2 kb) (S5 Fig).

We checked for misannotations by comparing each set of predicted proteins to the corresponding assembly using TBLASTN (*E*-value ≤1 × 10^−20^). This did not reveal any highly similar matches (discounting hits that overlapped with existing gene models), indicating that ‘missing’ *Rotaria* genes were not the result of poor gene prediction. However, the assemblies of all 4 species showed a large number of matches at lower similarity (30%–35% median identity at the amino acid level) (Fig 2C). These protein hits to putative noncoding regions may indicate pseudogenes, resulting either from degradation of coding regions following ancestral tetraploidisation or more recent duplications that have subsequently decayed and no longer encode functional proteins.

The structure of predicted genes also varied among species. The average intron length was 104 and 108 bp for *A*. *ricciae* and *A*. *vaga*, respectively, but up to 3 times longer in *R*. *macrura* and *R*. *magnacalcarata* (362 and 208 bp, respectively) (Fig 2D, Table 1; S6 Fig). Distributions of intron lengths showed 2 distinct classes, with the majority of introns falling in the range 30 to 100 bp but a substantial minority showing a higher variance around a much larger mean (inset of Fig 2D). The proportion of single-exon genes (SEGs) was substantially lower for *R*. *macrura* relative to other species, likely reflecting the lack of RNASeq guidance during annotation (Table 1).

The repeat content of bdelloid assemblies was measured following 2 approaches: (1) comparisons to known metazoan repeats, sampled from Repbase, and (2) comparisons to Repbase plus an additional library modelled ab initio from each assembly, using RepeatModeler (see Materials and methods). For (1), the relative abundances of TEs were low for all species, with the total proportion of interspersed repeats accounting for 1.2% of the assembly span for both *A*. *vaga* and *R*. *magnacalcarata*, 0.9% for *R*. *macrura*, and 0.8% for *A*. *ricciae* (S3 Data). Including simple and low-complexity repeats resulted only in modest increases, to 2.0%, 3.4%, 2.2%, and 3.0% for *A*. *ricciae*, *A*. *vaga*, *R*. *macrura*, and *R*. *magnacalcarata*, respectively. For (2), however, the inclusion of ab initio repeats resulted in considerably increased repeat content for all species but to a greater extent in *Rotaria* (16.8% for *A*. *ricciae*, 18.4% for *A*. *vaga*, 22.0% for *R*. *macrura*, and 27.6% for *R*. *magnacalcarata*). A large proportion of ab initio repeats were marked as ‘unclassified’, and their nature is yet to be determined (Fig 2B; S3 Data). The composition of bdelloid genomes with respect to genome size evolution is considered further below.

### Marked differences in intragenomic divergence among bdelloid species

**Fig 3:**
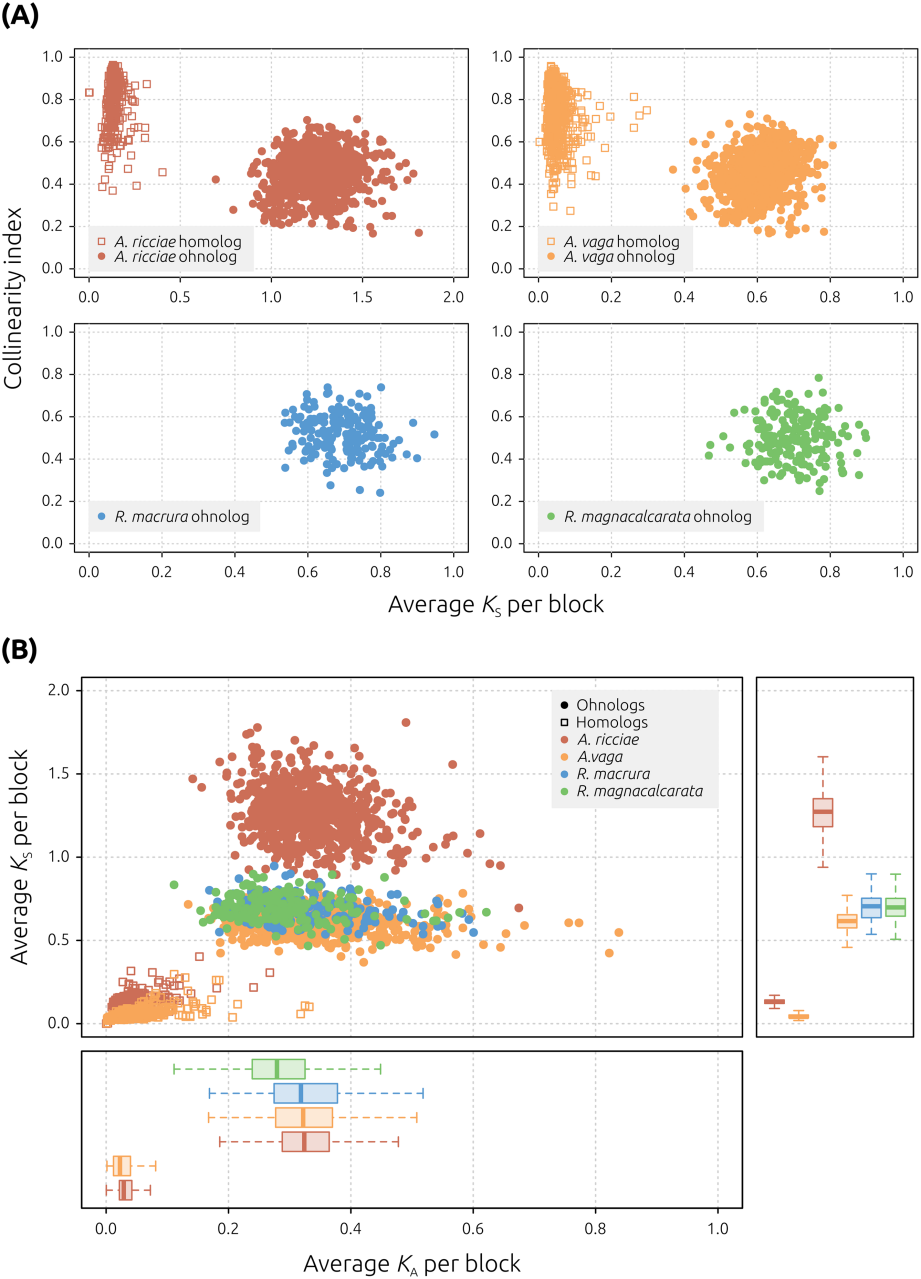
Collinearity in bdelloid genomes. (A) Collinearity versus synonymous divergence. Points represent collinear blocks of genes, plotted based on the average pairwise *K*_S_ between pairs of genes within the block (x-axis) and a ‘collinearity index’, defined as the number of collinear genes divided by the total number of genes within the genomic boundaries of that block (y-axis). Genes within *Adineta* species are clearly differentiated into 2 groups: homologs (low *K*_S_ and high collinearity; open squares) and ohnologs (high *K*_S_ and low collinearity; filled circles). In *Rotaria* species, however, genes within identified collinear blocks only show high *K*_S_ and low collinearity, equivalent to that observed between ohnologs in *Adineta*. Note the different x-axis limit for *A*. *ricciae*, reflecting a higher synonymous divergence between homologs in this species. (B) Synonymous versus nonsynonymous divergence per collinear block. Genes within homologous blocks (open squares) show a low rate of synonymous (*K*_S_) and nonsynonymous (*K*_A_) substitution. Homologous gene copies are not resolved in *Rotaria* assemblies. Genes within ohnologous blocks (filled circles, found in all species) show relatively higher rates of both *K*_S_ and *K*_A_. Right-hand panel shows elevated mean *K*_S_ in both *A*. *ricciae* homologs (0.14 ± 0.036 [SD] versus *A*. *vaga* = 0.05 ± 0.026) and ohnologs (1.27 ± 0.146 versus *A*. *vaga* = 0.61 ± 0.062; *R*. *macrura* = 0.70 ± 0.080; and *R*. *magnacalcarata* = 0.70 ± 0.078). This elevation is not observed for *K*_A_ (lower panel). Box-plots span the median (thick line), 50% of the values (box), and 95% of the values (whiskers).

Our assembly results show an apparent 2-fold difference in the number of genes encoded by *Adineta* species relative to *Rotaria* species, suggesting substantial differences in either ploidy or divergence patterns between bdelloid genera. To investigate the evolutionary relationships among genes within each species, we estimated nucleotide divergence and collinearity among separately assembled gene copies using MCScanX [50]. This analysis identifies collinear blocks of genes, defined as pairs of genomic regions that show conserved gene order (see Materials and methods). We plotted the average synonymous divergence (*K*_S_) between genes within each collinear block against a ‘collinearity index’, defined as the number of collinear genes divided by the total number of genes within a given block (following [28]). Both the *A*. *ricciae* reference and the (reannotated) *A*. *vaga* 2013 assembly showed a clear delineation of genes into both homologs (low *K*_S_ and high collinearity) and ohnologs (high *K*_S_ and low collinearity) (Fig 3A; S4 Data), as has been observed previously for *A*. *vaga* [28]. The number of *A*. *ricciae* genes that form homologous collinear blocks is 36,593 (73.4% total genes); for *A*. *vaga*, it is 37,061 (64.5%) (S2 Table). Comparisons between *Adineta* species show approximately half as many homologous collinear blocks in *A*. *ricciae* relative to *A*. *vaga* (475 versus 905), but these contain twice as many genes (median 24 versus 11). Therefore, the extent to which we have successfully captured homologous gene copies in *A*. *ricciae* appears to be at least equivalent to that for *A*. *vaga*. Strikingly, however, only ohnologous relationships are inferred in *Rotaria* genomes: collinear blocks composed of homologous genes are not observed (Fig 3A). Comparisons of ohnologous blocks across all species also suggest that the extent of ohnologous collinearity is higher in *Adineta* species than in *Rotaria* species (more ohnologous blocks comprising a greater proportion of genes), notwithstanding confounding factors such as differences in the level of assembly fragmentation (S2 Table).

Assuming that the ancestor of extant *Rotaria* lineages was also tetraploid [25], the apparent ‘loss’ of homologous copies in *Rotaria* species may be caused by either (1) the genuine loss of homologous gene copies from *Rotaria* genomes—resulting in a shift from tetraploidy to highly diverged diploidy—or (2) extremely low levels of divergence between *Rotaria* homologs, such that the majority of homologous sites are identical and cannot be separately assembled (i.e., are collapsed). To differentiate between these hypotheses, we characterised patterns of nucleotide polymorphism and read coverage across each genome, as has been used to investigate the genomes of other polyploid or asexual species [51–55]. Widespread assembly collapse of homologous regions should result in single-nucleotide polymorphisms (SNPs) with a frequency around 50% and a total coverage (read depth of reference plus alternative bases) that is approximately equal to the genome-wide average, analogous to collapsed heterozygous sites in a segregating diploid genome (e.g., see Fig 2A of [42]). These patterns are not predicted under the hypothesis of gene loss in an uncollapsed assembly (i.e., all haplotypes separately assembled), where SNPs may arise in repetitive regions (TEs, tRNAs, low-complexity regions, etc.) but are unlikely to show a frequency of 50% or consistent read depth.

**Fig 4:**
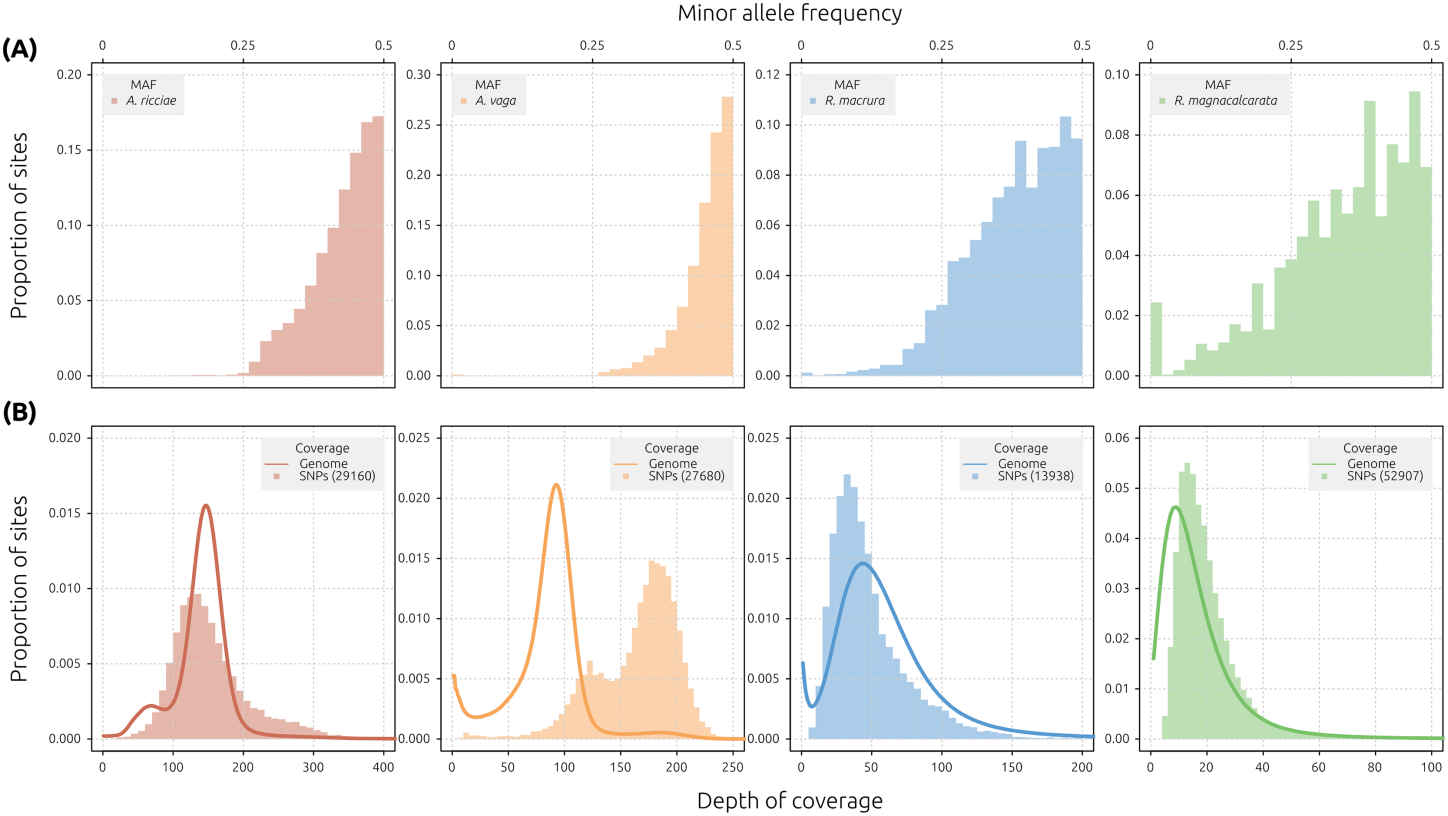
Distributions of MAF and read coverage. (A) Folded MAF spectra for detected SNPs in the reference genomes of *A*. *ricciae* (red), *A*. *vaga* (orange), *R*. *macrura* (blue), and *R*. *magnacalcarata* (green) are distributed around a mode of 0.5 in all species. (B) Read and SNP coverage distributions. In each plot, the bar histogram represents the distribution of read coverage at SNP sites only, while the overlayed line shows the distribution of read coverage across all sites in the genome. The y-axes indicate proportion sites with given depth of each category (i.e., peak heights are relative to each category). The number of SNPs contributing to the bar histogram is indicated in parentheses (see legend). The cause of the secondary peak in SNP depth (at approximately 125x) for *A*. *vaga* (library ERR321927) is unknown. MAF, minor allele frequency; SNP, single-nucleotide polymorphism.

Reads were mapped to the reference assembly of each species, using single-clone (*A*. *ricciae* and *A*. *vaga*) or single-individual (*R*. *macrura* and *R*. *magnacalcarata*, whole genome amplified [WGA]) libraries. High-quality biallelic SNPs showing a minor allele frequency (MAF) distributed around 50% were detected in all assemblies, indicating at least partial collapse in all cases (Fig 4A, S5 and S6 Data). The relative platykurtosis observed in *Rotaria* species may be an artefact of WGA (inflation of low-frequency SNPs) or lower coverage in general (S7 Fig). In *A*. *vaga*, the majority of sites (approximately 76%) show coverage around 90x, representing separately assembled regions, with a minor peak at 180x, representing collapsed regions (Fig 4B; S8 Fig). The majority of SNPs occur in regions of 180x coverage, as would be expected under the scenario of localised assembly collapse [28]. For both *R*. *macrura* and *R*. *magnacalcarata*, however, read depth at SNP sites (reference plus alternative alleles) varied in concert with the genome-wide coverage distribution (Fig 4B). These patterns indicate that the majority of SNPs occur in collapsed regions, supporting the hypothesis of widespread assembly collapse in *Rotaria* species.

A different pattern is observed in *A*. *ricciae*, however. Here, a small proportion of sites (11%) are distributed around a peak at 75x coverage, which presumably represents the 1-fold coverage value, but the majority of sites (81%) show 150x coverage and are thus presumably 2-fold covered (i.e., present in double copy) (Fig 4B; S9 Fig). Furthermore, SNP depth is unimodal and is centred on the 150x coverage peak, indicating that the majority of variant sites occur in regions of putative 2-fold coverage. Given the successful capture of the majority of homologous gene copies in *A*. *ricciae* (approximately 73%, S2 Table), we infer that these conflicting signals are likely derived from another source of coverage heterogeneity that is unrelated to homologous collapse. This is unlikely to be due to an additional whole-genome duplication in *A*. *ricciae*, given that both *Adineta* species have 12 chromosomes [56,57], but may be caused by other phenomena that affect DNA stoichiometry at the level of either the genome (e.g., segmental or partial genome duplications) or the sample itself (e.g., endopolyploidy [58] or cryptic population structure) (S1 Text, S10 Fig). Further investigations of the *A*. *ricciae* genome are required to test these hypotheses.

*A*. *ricciae* displayed a further difference from other bdelloid genomes: a clear elevation in *K*_S_, both for homologs (compared to *A*. *vaga*; mean *K*_S_^Ar^ = 0.135 versus *K*_S_^Av^ = 0.05; *t* = 47, *P* < 0.01) and for ohnologs (e.g., mean *K*_S_^Ar^ = 1.267 versus *K*_S_^Av^ = 0.613; *t* = 124, *P* < 0.01) (Fig 3B; S3 Table). No such elevation was observed in the rate of nonsynonymous substitution in *A*. *ricciae*, compared to the other species. However, the *A*. *ricciae* genome also shows the highest GC content of the 4 species (approximately 5% higher than *A*. *vaga*, and 3% to 4% higher than either *Rotaria* species). Therefore, one explanation for the increase in *K*_S_ may be selection for increased GC content in *A*. *ricciae*, with continued purifying selection at nonsynonymous sites [59].

### Homologous divergence in *Rotaria* species is lower than allelic divergence in most sexual species

**Fig 5:**
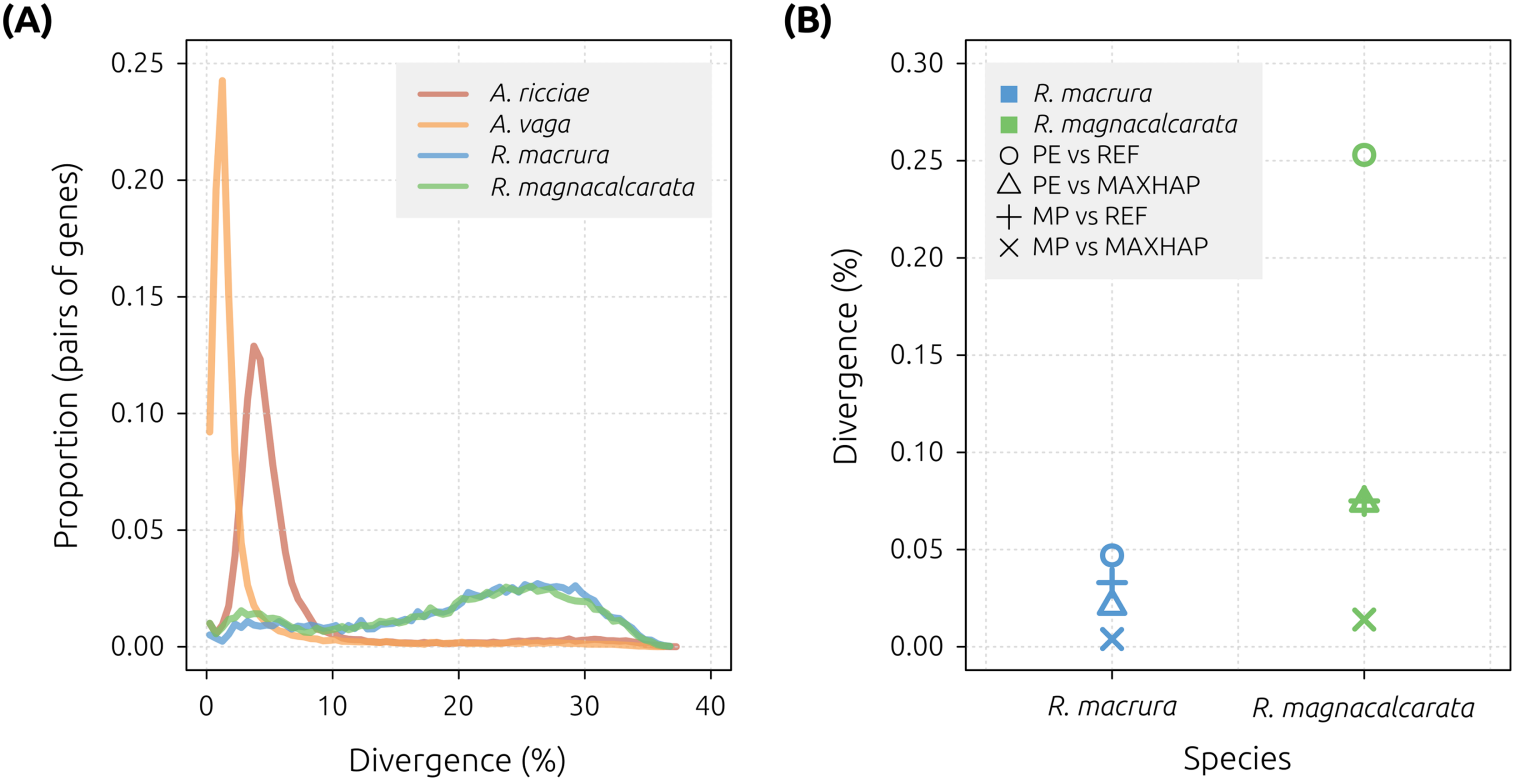
Estimates of intragenomic divergence. (A) Distribution of sequence identity (expressed as a divergence, i.e., proportion of nonidentical sites) for the top non-self BLAST hits from intragenomic comparisons for each species, showing highly similar gene copies within *Adineta* genomes but not within *Rotaria* genomes. Median values are 4.55% (*A*. *ricciae*, red), 1.42% (*A*. *vaga*, orange), 22.5% (*R*. *macrura*, blue), and 21.7% (*R*. *magnacalcarata*, green). (B) Homologous divergence estimated from SNP counts in coding regions in REF and MAXHAP assemblies for *R*. *macrura* and *R*. *magnacalcarata*. Mean estimates are 0.026% for *R*. *macrura* and 0.104% for *R*. *magnacalcarata*. MAXHAP, maximum haplotype; MP, mate pair; PE, paired end; REF, reference; SNP, single-nucleotide polymorphism.

In the *A*. *ricciae* reference and *A*. *vaga* 2013 assemblies, the majority of homologs were separately assembled, allowing for the identification of homologous gene copies and the estimation of their sequence divergence using a BLAST-based approach [51,55]. The median divergence between separately assembled homologous gene copies was 4.55% (mode = 3.75%) in *A*. *ricciae* and 1.42% (mode = 1.25%) in *A*. *vaga* (in agreement with [28]) (Fig 5A). However, these estimates may be inflated because they fail to consider homologous regions with low divergences that are collapsed. Alternative estimates of homologous divergence based on SNPs detected in the collapsed *A*. *vaga* assembly were correspondingly lower (0.955% and 0.788%, based on alignment of libraries ERR321927 and SRR801084, respectively) (S5 Data).

Exploration of alternative assembly strategies, which aimed to minimise as much as possible the phenomenon of assembly collapse, did not result in the separate assembly of *Rotaria* homologous regions (S1 Data), indicating substantially lowered homologous divergence relative to *Adineta*. To estimate the divergence between collapsed *Rotaria* homologous gene copies, we instead counted the number of SNPs occurring in coding regions in each assembly. Based on single-individual, WGA mate-pair libraries aligned to the *R*. *macrura* and *R*. *magnacalcarata* reference assemblies, a total of 13,115 and 36,594 SNPs were detected across 40.0 and 40.3 Mb of coding sequences (CDSs), respectively (S5 and S6 Data). Assuming that all detected SNPs are the result of homologous collapse, an upper limit for the divergence between homologs is estimated at 0.033% and 0.075% for *R*. *macrura* and *R*. *magnacalcarata*, respectively (Fig 5B).

These results indicate that homologous divergence in nondesiccating *Rotaria* species is at least an order of magnitude lower than that observed in anhydrobiotic *Adineta* species. This contradicts hypotheses that emphasise the role of desiccation in shaping patterns of divergence in bdelloid genomes. If the rate of desiccation-induced DSB repair is positively correlated with the rate of gene conversion, a lower level of homologous divergence is expected in species with higher rates of desiccation. In fact, we observe the opposite: divergence between homologs in nondesiccating *Rotaria* species is considerably lower than in *A*. *ricciae* (median 4.6%), *A*. *vaga* (1.4%) (here and [28]), and *Philodina roseola* (3% to 5%) [24], all of which are capable of anhydrobiosis. Across sexual eukaryotes, estimates of allelic divergence range from about 0.01% to 8% [60]. Therefore, none of these bdelloid species is beyond the range of observed values for sexual taxa, although some fall near the extremes of this distribution (e.g., *Rotaria* are towards the lower end, in contrast to *A*. *ricciae* and *P*. *roseola*).

How can we reconcile theory with these observations? The simplest explanation is that homologous divergence is unlinked to desiccation and that the observed differences are instead reflective of underlying phylogeny. Alternatively, it may be that homogenisation between homologous gene copies in *Rotaria* is not caused by desiccation-induced DSB repair but by gene conversion arising during a different process, such as mitotic crossing over [61–63]. In the yeast *Saccharomyces cerevisiae*, for example, various forms of mitotic recombination can produce tracts of gene conversion many kilobases long, often initiated from DNA nicks that are subsequently processed into DSBs [64–66]. Such processes may be especially pronounced and irreversible in asexuals: rapid loss of heterozygosity is observed in recent asexual lineages of the water flea *Daphnia pulex*, in which high rates of initial heterozygosity in hybrid asexual lineages are rapidly eroded via gene conversion and hemizygous deletion, which may ultimately limit their longevity [67].

However, this begs the question: why should the same homogenising mechanisms not operate in desiccation-tolerant species? One possibility is that desiccation-tolerant species have low or negligible background rates of gene conversion while hydrated, thanks to selection for highly effective DNA repair and error-checking mechanisms imposed by environments that desiccate on a regular basis. Such a repair system might faithfully prevent loss of diversity in the context of mitosis, even while occasional gene conversion remains an unavoidable consequence of the more demanding repairs required after desiccation. Alternatively, perhaps similar homogenising forces do indeed operate in desiccation-tolerant rotifers but are counteracted by mutations generated during repair of desiccation-induced DSBs, whose net effect is to sustain high rates of homologous divergence [68]. Positive evidence for a link between desiccation and DSBs in bdelloids is currently limited to experiments in a single species, *A*. *vaga* [31], and evidence from other anhydrobiotic taxa is mixed [32,33,35]. Further work on DNA integrity and genome evolution in bdelloids is needed to address these divergent predictions. A final explanation that cannot be entirely excluded is that low homologous divergence in *Rotaria* genomes results from cryptic sexual reproduction, constrained by small population sizes (i.e., inbreeding), although no males have so far been detected in *Rotaria* or any other bdelloid [69].

### Architectural signals of long-term ameiotic evolution are lacking in *A*. *ricciae*

The *A*. *vaga* 2013 assembly (accession GCA_000513175.1) showed a number of unusual structural genomic features, including breaks in homologous collinearity, physical linkage of homologous genes (i.e., encoded on the same scaffold), and genomic palindromes of the form g1_A_, g2_A_, g3_A_…g3_B_, g2_B_, g1_B_, where A and B denote homologous copies of genes g1, g2, and g3. Such features would result in chromosomes that cannot be decomposed into haploid sets and thus imply a genome architecture that is incompatible with conventional meiotic pairing and segregation, as might be predicted under the hypothesis of long-term asexuality [28].

**Fig 6:**
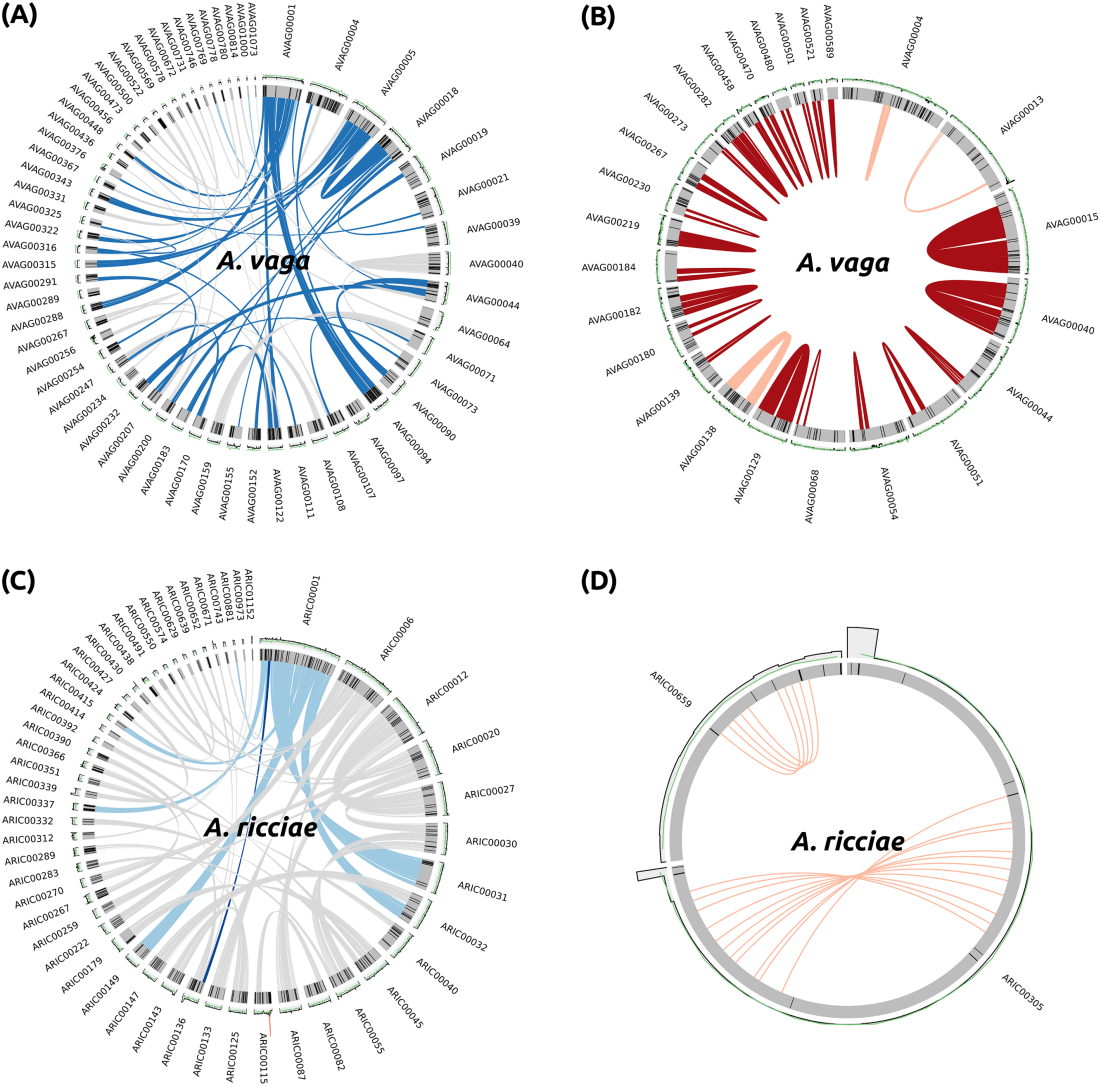
Unusual genomic features are not detected in *A*. *ricciae*. Genomic scaffolds are shown as grey bars, GC content (green line), and read coverage (grey histogram) averaged over 5-kb bins are shown above each scaffold. Black lines within scaffolds represent gaps (Ns) introduced during scaffolding. (A) Example of collinearity breaks in *A*. *vaga* 2013 assembly scaffold AVAG00001. Links between homologous blocks are shown in light blue; downstream connections are shown in grey. Collinearity breaks are shown in dark blue. (B) The majority of homologous blocks encoded on the same scaffold in the *A*. *vaga* 2013 assembly are palindromes (dark red); tandem repeats are shown in pink. (C) Example of a single collinearity break on *A*. *ricciae* scaffold ARIC00001. (D) No genomic palindromes detected in *A*. *ricciae*. Only 2 homologous blocks were found on the same scaffold (ARIC00305 and ARIC00659), both arranged as tandem repeats. GC, guanine-cytosine.

To test for such structures in other bdelloid genomes, we first analysed the reannotated *A*. *vaga* 2013 assembly. A total of 298 breaks in collinearity (32.9% of 905 homologous blocks) were detected (an example is shown for scaffold AVAG00001 in Fig 6A). In addition, 25 homologous blocks were encoded on the same genomic scaffold, 2 as tandem arrays and 23 as palindromes (Fig 6B). Thus, our detection methods were able to recover the same signals of ameiotic evolution reported by Flot et al. (2013) [28], for the same *A*. *vaga* assembly. The method of Flot et al. (2013) was to construct contigs from Roche 454 Titanium and GS-FLX data using the MIRA assembler [70], followed by correction and scaffolding using high-coverage Illumina paired-end and mate-pair data (section C1 of [28] supplement). We attempted to reassemble the *A*. *vaga* paired-end Illumina data independently (incorporating both mate-pair and 454 data for scaffolding) using a variety of established short-read assemblers. However, this consistently resulted in highly fragmented assemblies (e.g., N50 of approximately 1 to 2 kb), except when allowing for the collapse of homologous regions (S1 Data). The lack of contiguity in *A*. *vaga* maximum haplotype assemblies precluded us from using alternative assembly approaches to investigate the features detected in the 2013 assembly. A lack of separately assembled homologous gene copies in either *Rotaria* species similarly precluded structural analysis.

The closely related species *A*. *ricciae*, however, showed both high assembly contiguity and a majority of separately assembled homologous gene copies. For this species, we detected only 8 collinearity breaks (1.7% of 466 homologous blocks) between homologs (an example is shown in Fig 6C), in contrast with hundreds inferred from the *A*. *vaga* 2013 assembly. Assuming that the *A*. *ricciae* and *A*. *vaga* 2013 assemblies are structurally accurate and that the homologous gene copies captured in both assemblies reflect the ancestral tetraploidisation common to all bdelloids, collinearity appears to be markedly more conserved in *A*. *ricciae* relative to *A*. *vaga*. However, many of the detected breaks in both species span regions separated by scaffold gaps (Ns introduced during the joining of contigs), suggesting that at least some detected collinearity breaks may be the result of scaffolding errors despite requisite care during assembly (Fig 6A and 6C). For example, an unscaffolded *A*. *ricciae* assembly showed only a single break in collinearity, although the increased fragmentation of this assembly (N50 = 18.7 kb) may limit our ability to detect such breaks. We did not detect any cases of homologous genes arranged as palindromes in *A*. *ricciae*: only 2 cases of linked homologous blocks were detected, both tandem repeats (Fig 6D).

Overall, these results suggest that certain unusual genomic features, previously interpreted as positive signatures of long-term ameiotic evolution in *A*. *vaga* [28], are largely absent from the closely related *A*. *ricciae* (and remain untested in other bdelloids). These patterns may reflect true differences between *Adineta* species, although no marked dissimilarity in karyotype is evident [56,57]. Alternatively, they may be either false-positive or false-negative artefacts of applying alternative assembly methodologies to complex genomes with different patterns of intragenomic divergence. Evidence from other taxa is limited. For example, similar features have been reported in the recently assembled genomes of the parthenogenetic springtail *Folsomia candida* [71] and in certain apomictic species of *Meloidogyne* root-knot nematodes [72]. However, these involved only small proportions of each respective genome, and the latter study used the same assembly approach that was applied to *A*. *vaga* [72]. Furthermore, recent investigations of genome evolution in asexual, nondesiccating *Diploscapter* nematodes have revealed a high degree of collinearity among homologous genes, despite high levels of divergence (approximately 4%, thus similar to *A*. *ricciae*) [54,73]. These data suggest that transitions to asexuality do not necessarily lead to the erosion of collinearity. Similar structural variants are also detected in many sexual organisms—for example, humans [74,75], cichlid fishes [76], and cows [77]—and may involve translocations or duplications that are many kilobases in length. Therefore, further work is required to improve and validate assembly contiguity of bdelloid genomes and to ascertain the evolutionary significance of these features.

### Variation in genome size driven by expansion of noncoding elements in *Rotaria* species

Although variation in genome size among bdelloids has been inferred previously based on cytofluorometry of oocytes [78], there are some inconsistencies between reported values that indicate possible errors (see S2 Text for details). Based on our assembly results, estimations of global genome properties such as total span and gene number can be estimated bioinformatically, using both kmer- and assembly-based approaches. The maximum haplotype assembly for *A*. *ricciae* was approximately 201 Mb in length encoding 63,000 genes, while reannotation of the partially collapsed 217-Mb *A*. *vaga* 2013 assembly showed approximately 67,000 genes. While maximum haplotype assemblies for *A*. *vaga* were highly fragmented (and therefore poorly annotated), a collapsed *A*. *vaga* assembly, reduced to 109 Mb, encoded 31,600 genes. Notwithstanding potential complexities of coverage heterogeneity in the *A*. *ricciae* data (discussed above), these values suggest that the full complement of genes in *A*. *ricciae* may be in the region of 60,000 to 65,000 genes across a total span of about 200 Mb (S11 Fig). The genome of *A*. *vaga* is likely to be of approximately equivalent size [28]. The largely collapsed reference assemblies of *R*. *macrura* and *R*. *magnacalcarata* showed about 25,000 and 35,000 genes, respectively, and thus are in broad agreement with observations from *Adineta*. This also implies that the total genome size for *Rotaria* is in the region of 400 to 500 Mb, assuming the majority of sites are in double copy (S11 Fig), and indicates that the genomes of *Rotaria* species may be considerably larger relative to *Adineta* species.

What mechanisms might explain these observed differences? Based on comparisons to known metazoan TEs from Repbase, the abundance of TEs and low-complexity repeats was low in all species, suggesting that expansions of known TEs or simple repeats in the *Rotaria* lineage is unlikely to be a major driver. However, the inclusion of the largely unclassified ab initio repeats did result in a marked increase in total repetitive sequences for all species (17%, 18%, 22%, and 28% for *A*. *ricciae*, *A*. *vaga* 2013, *R*. *macrura*, and *R*. *magnacalcarata*, respectively). The relative increase is greatest in the *Rotaria* species, suggesting that a substantial fraction of the *R*. *macrura* and *R*. *magnacalcarata* reference assemblies are covered by repeats whose exact nature remains to be elucidated. In addition, average intron sizes in *Rotaria* genes are longer (by at least 100%), driven primarily by an increase in the number of long introns. Intriguingly, a similar association between desiccation tolerance and genome ‘compaction’ has been observed in tardigrades: *Hypsibius dujardini* has a genome size of 104 Mb and only survives desiccation under certain conditions, whereas *Ramazzottius varieornatus* has a much smaller genome size of 56 Mb and is capable of rapid anhydrobiosis [34,79]. Future sampling of more phylogenetically independent comparisons of desiccating and nondesiccating species is needed to test these ideas.

### Genome content analysis substantiates high levels of HGT, low numbers of TEs, and presence of meiosis genes

**Fig 7:**
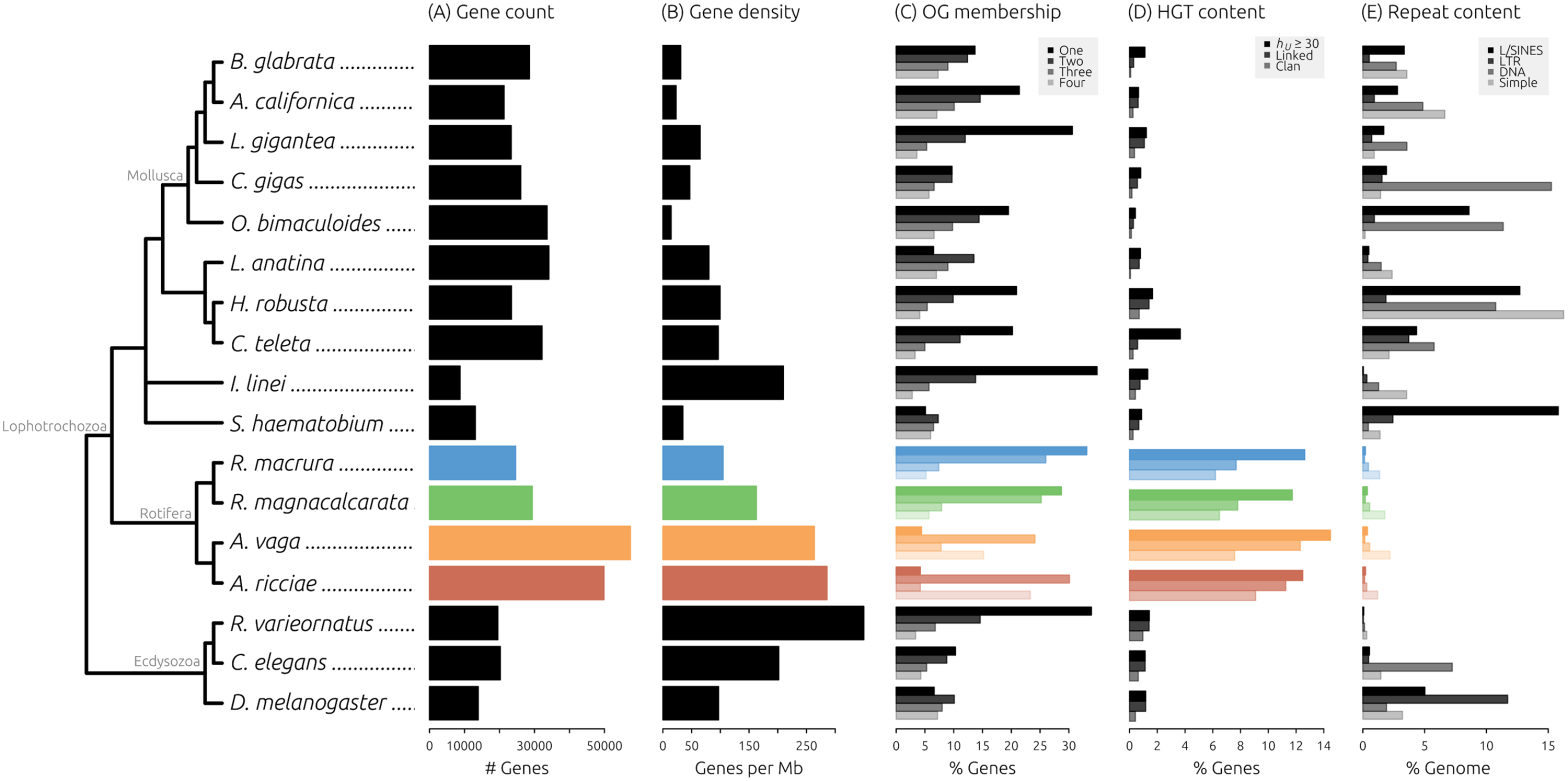
Bdelloid genome characteristics compared to other metazoans. Phylogenetic relationships among 17 protostome species are shown on the left, major groups are marked in grey. (A) Gene count, defined as the number of protein-coding genes in each genome (taken from Ensembl Metazoa [90] or from the relevant literature). (B) Gene density, defined as the number of genes divided by the genome span (inferred from assembly). (C) Same-species OG membership, with bars from top to bottom representing 1-, 2-, 3-, and 4-member clusters. (D) HGT content, with bars from top to bottom representing the proportion of genes with *h*_*U*_ ≥30 for each species (HGT_C_), in physical linkage with a known metazoan gene (‘Linked’), and with clan membership to nonmetazoan genes from phylogenetic analyses (‘Clan’). (E) Repeat content, with bars from top to bottom representing the proportion of each genome covered by LINE/SINEs, LTR elements, DNA elements, and simple/low-complexity repeats. HGT, horizontal gene transfer; HGT_C_, HGT candidates; LINE, long interspersed element; LTR, long terminal repeat; OG, orthologous group; SINE, short interspersed element.

To better understand how bdelloid genomes compare to those of other metazoans, we characterised an additional 13 species from across the Protostomia (S4 Table) based on genome size, gene density, patterns of orthologous gene clustering, HGT content, and repetitive sequence content (Fig 7, S12 Fig, S5 and S6 Tables). Our comparison included a broad taxonomic range of species from different ecological niches, including molluscs [80–83], annelids [81], the platyhelminth *Schistosoma haematobium* [84], the desiccation-tolerant tardigrade *R*. *varieornatus* [34], the orthonectid intracellular parasite *Intoshia linei* [85], and chromosomal-level reference genomes for *Caenorhabditis elegans* [86] and *Drosophila melanogaster* [87]. Phylogenetic relationships among species were not estimated directly but inferred from the literature [85,88,89].

We assessed the extent of horizontal transfer into protostome genomes using both sequence comparison and phylogenetic approaches. The extent to which HGT contributes to the genomes of multicellular eukaryotes is controversial. For example, a recent claim of 17% nonmetazoan genes encoded in the genome of the tardigrade *H*. *dujardini* was later shown to be derived mostly from contaminating non–target organisms [91–94]. Nonetheless, a high proportion of genes from a variety of nonmetazoan sources has consistently been inferred in bdelloid genomes from a range of independent data, including fosmid sequences [26], transcriptomes [27,41], and whole-genome data [28]. To measure the level of horizontal transfer, we developed an HGT assessment pipeline that uses both sequence comparison and phylogenetic approaches to build a body of evidence for the foreignness of each predicted gene. Our goal was not to unequivocally assert the evolutionary history of individual genes but rather to apply these tests consistently across the set of animal genomes as a fair comparison for estimating HGT.

Our initial screen identified 6,221 (12.5%), 8,312 (14.5%), 3,104 (12.6%), and 3,443 (11.7%) genes from *A*. *ricciae*, *A*. *vaga*, *R*. *macrura*, and *R*. *magnacalcarata*, respectively, as HGT candidates (HGT_C_) (Fig 7D, S7 Data). These values are substantially higher than the proportion of HGT_C_ observed in any other protostome species included in this analysis, using the same pipeline and thresholds (the highest proportion of HGT_C_ for a non-bdelloid was 3.6%, for the annelid worm *Capitella teleta*). This is also noticeably higher than estimates based solely on the Alien Index. For each HGT_C_, we then assessed (1) the presence of predicted introns, (2) scaffold linkage to another gene of unambiguous metazoan origin, (3) presence on a scaffold that encodes a high HGT_C_ proportion that might indicate contamination, (4) membership within a ‘clan’ of nonmetazoan orthologs, and (5) monophyly of the HGT_C_ with all present nonmetazoan orthologs to the exclusion of all metazoan orthologs (see Materials and methods). Testing for clan membership with nonmetazoan orthologs reduced the proportion of HGT_C_ to 9.1%, 7.6%, 6.2%, and 6.5% for the 4 bdelloids, compared with <1% for all other species (S7 Data). The final test was not applicable for the majority of HGT_C_ because metazoan orthologs were often not detected; thus, the number of genes that additionally showed evidence for monophyly with nonmetazoan orthologs was 189, 190, 111, and 82 for *A*. *ricciae*, *A*. *vaga*, *R*. *macrura*, and *R*. *magnacalcarata*, respectively, and was reduced to a handful or 0 in all other species. Sequential BLAST analysis of HGT_C_ ‘clan’ genes showed that the majority (approximately 80%) were found in all 4 bdelloids, suggesting that most detected HGT_C_ genes are of ancient origin (S13 Fig). Correspondingly, many HGT_C_ genes were also found as either pairs or quartets (for *A*. *ricciae* and *A*. *vaga*) or as singletons or pairs (for *R*. *macrura* and *R*. *magnacalcarata*) (S13 Fig).

These comparisons support previous findings of a high proportion of nonmetazoan genes in bdelloid genomes [26,28,41,95]. Compared to other metazoans, and at all levels of scrutiny, the 4 bdelloid genomes analysed here showed a substantially greater proportion of genes from nonmetazoan sources than do any other species in our comparison. Our results confirm a substantial proportion of foreign genes in the nondesiccating *Rotaria* genomes, in agreement with recent findings based on transcriptomes [41]. Our assessment also showed very low levels of HGT (approximately 1%) into the genome of the anhydrobiotic tardigrade *R*. *varieornatus*, in agreement with recent estimates [34,79]. In addition, recent genome investigations of the anhydrobiotic chironomid insect *P*. *vanderplanki*, which experiences a large number of DNA breakages during desiccation [35], also did not reveal an elevated rate of HGT [96]. Taken together, these findings bring into question the association between anhydrobiosis and elevated rates of HGT that previously has been suggested for bdelloids [26,28,30,31,95]. One explanation may be that differences in HGT content reflect species-specific differences in the mechanism of anhydrobiosis, in combination with particular ecological properties of each species. Further comparative work is thus required to elucidate any relationship between anhydrobiosis and horizontal transfer.

An alternative possibility is that HGT content in bdelloids does not reflect a deviation in the rate of import of foreign genes but an increased rate of retention arising from their putative longstanding asexuality. Based on transcriptome data from *Rotaria* species, Eyres et al. (2015) estimated the rate of gain to be low in absolute terms, on the order of approximately 10 HGT gains per lineage per million years [41]. Perhaps this is a typical background rate of import for organisms with similar ecological and physiological properties to bdelloid rotifers, but acquired genes are able to persist for longer in an ameiotic background given the lack of mechanisms such as segregation and unequal crossing over that would otherwise remove them. If so, foreign genes incorporated by asexuals, even if initially deleterious, might persist over the extended timescales necessary for domestication. The high proportion of nonmetazoan genes accumulated in bdelloid genomes may therefore owe more to a long-term lack of meiotic sex than to anhydrobiosis.

We also quantified the abundance of TEs and low-complexity repeats in each animal genome. We chose to focus on the quantification of known repeats and thus did not perform ab initio repeat modelling for non-bdelloid species. There was considerable variation in TE abundance among species (Fig 7E), with the total proportion of genome covered by interspersed repeats varying from 0.3% in the tardigrade *R*. *varieornatus* to 27.5% in the oyster *Crassostrea gigas* (S3 Data). The relative abundance of different classes of repeats, including long interspersed elements (LINEs), short interspersed elements (SINEs), long terminal repeats (LTRs), and DNA elements, also differed greatly among taxa, as did the amount of simple and low-complexity repeats. The proportion of total repeats (TEs plus low-complexity repeats) ranged from 0.6% in *R*. *varieornatus* to 42% in the annelid worm *Helobdella robusta*. All 4 bdelloid species display a low abundance of TEs, in agreement with previous findings [23–25,28,97]. However, 2 other species also show low levels of TEs: *I*. *linei*, an intracellular parasite of marine invertebrates with a highly reduced genome (42 Mb) [85], and *R*. *varieornatus*, also with a relatively small genome (56 Mb) [34]. In fact, *R*. *varieornatus* encodes the fewest TEs of the species analysed here (0.6% as a proportion of assembly span), followed by the 4 bdelloids (2%–3%). These estimates of TE abundances in *I*. *linei* and *R*. *varieornatus* are substantially lower than the total repeat content of these genomes (28% and 20%, respectively), which includes a high proportion of ab initio repeats (inferred directly from the assembled nucleotides) marked as ‘unclassified’ (accounting for approximately 18% and approximately 19% total repeats, respectively [79,85]), matching our finding of higher ab initio repeat content in bdelloids. Additional work is required to elucidate the nature of these unclassified repeats in bdelloids and in other taxa.

What evolutionary forces may explain the low abundance of TEs in these species? Asexuality and anhydrobiosis have both previously been posited as factors contributing to the low number of TEs in bdelloid rotifers. For example, under long-term asexual evolution, TEs may proliferate freely within a genome and thus drive that lineage to extinction (an extension of Muller’s ratchet) or become lost, domesticated, or otherwise silenced [30,98–100]. Frequent cycles of desiccation and rehydration may also favour the evolution of reduced repeat content, via selection against deleterious chromosomal rearrangements brought about by ectopic recombination of TEs during the repair of DSBs [29,30].

Our comparisons did not detect any substantial variation in the abundance of known TEs between desiccating (1.2% and 0.8% for *A*. *ricciae* and *A*. *vaga*, respectively) and nondesiccating (0.9% and 1.2% for *R*. *macrura* and *R*. *magnacalcarata*, respectively) species, despite a considerable increase in the inferred genome size of *Rotaria* species. Moreover, the proposed mechanism involving desiccation relies on DSB repair during rehydration, a process which is presumably limited in the aquatic species *R*. *macrura* and *R*. *magnacalcarata* and may also not apply in the case of *R*. *varieornatus*, whose DNA is protected during anhydrobiosis [34]. However, the vast majority of bdelloid rotifers are resistant to desiccation, suggesting that anhydrobiosis was probably the ancestral state [17]. Therefore, it may be that TEs and other repeats were already largely eradicated in the most recent common ancestor to nondesiccating *Rotaria* species, prior to their adaptation to a fully aquatic lifestyle and loss of anhydrobiosis.

Finally, we also tested for the presence of a suite of 41 sex-related genes [101] in bdelloids using both TBLASTN (comparing to the genome) and HMMER (comparing to the proteome). Tested genes included 11 associated with meiosis, 19 involved in recombinational repair, 6 involved in DNA damage detection, 4 involved in DSB repair via nonhomologous end-joining, and 1 involved in bouquet formation (S8 Data). A positive match using TBLASTN and/or HMMER was recorded in at least 1 bdelloid species for all tested genes (40 of 41; 98%) with the exception of *RED1*, which is involved in crossover regulation and was not detected in any bdelloid at any significance threshold (S8 Data). However, *RED1* was not detected in *D*. *melanogaster* and only as a poor match in *C*. *elegans* and thus may represent an ancestral loss that predates the bdelloids. Overall, these findings suggest that bdelloids do encode the majority of genes involved in meiosis and sex-related functions. However, the presence of these genes does not necessarily indicate the presence of sex or meiosis because they are likely to be retained for other functions related to homologous recombination and DSB repair [102].

## Concluding remarks

The bdelloid rotifers have drawn attention because 2 features of their life history are remarkable among metazoans: their apparent ancient asexuality and their ability to withstand desiccation at any life stage. In this work, we have generated whole-genome sequence data for 3 additional bdelloid species with the overall aim of assessing hypotheses regarding the contributions of asexuality and anhydrobiosis to their genome evolution.

We find that both desiccating and nondesiccating species are ancestrally tetraploid, in agreement with previous work, but that homologous divergence in nondesiccating *Rotaria* species is substantially lower than that observed in anhydrobiotic *Adineta* species and may be low even compared to estimates of allelic heterozygosity from sexual eukaryotes. This finding runs counter to predictions based on current hypotheses regarding the genomic effects of desiccation and thus requires a reevaluation of the causes and consequences of intragenomic interactions between bdelloid homologs. Comparisons of genome architecture revealed that a number of unusual genome features posited as evidence of long-term ameiotic evolution in *A*. *vaga* were largely absent from the closely related species *A*. *ricciae*, for which a comparable assembly is now available. In addition, we find that bdelloids encode the majority of genes that are required for meiosis and syngamy in sexual taxa but emphasise that the precise function of these genes in bdelloids is currently unknown.

We reconfirm previous reports that bdelloids encode a high proportion of nonmetazoan genes. Here too, a role for desiccation tolerance had been hypothesised. We find that high HGT content is a potentially unique feature of bdelloid genomes among animals, but comparisons to other desiccation-tolerant taxa raise questions about the role of anhydrobiosis. Our extensive assembly results also allow for a refinement of the global parameters of bdelloid genomes and suggest substantial genome size differences between genera. The phylogenetic nonindependence of our comparative analysis currently precludes any certainty in linking these observed trends to desiccation tolerance. Further elucidation will be possible when data for anhydrobiotic species within *Rotaria* become available in the future. Overall, we conclude that many features of the bdelloid genomes analysed here are not markedly inconsistent with those found in sexual taxa, except for the remarkably high prevalence of HGT.

Finally, we hope that our approach may offer useful guidance for future studies involving the de novo assembly of non–model organisms with complicated genome characteristics from complex raw data. Our goal was to explore the assembly parameter space for each dataset, taking into consideration a number of potential confounding factors including polyploidy, intragenomic divergence, and sample polymorphisms. Our assembly results showed good contiguity and gene-completeness metrics, indicating a high level of overall quality. Nonetheless, we reiterate the caution that a full understanding of genome architecture and evolution in bdelloid rotifers will be possible only with highly contiguous, chromosome-level assemblies, towards which future efforts will be directed.

## Materials and methods

### Rotifer culture and sampling

Clonal cultures of *A*. *ricciae* [103] rotifers were grown as previously described [27,104–106]. Briefly, rotifers were grown in T75 tissue culture flasks (Nunc) with 15 to 25 ml ddH_2_O and fed twice a week with 10 μl of either bacteria (*Escherichia coli* TOP10 [ThermoFisher] in water) or a solution of yeast extract and peptone (2.5% w/v each). Approximately 50,000 rotifers were starved overnight before collection and harvested by centrifugation at 10,000 g for 5 minutes before treatment according to the relevant DNA or RNA extraction protocol. A starter culture for *R*. *macrura* was generated from approximately 100 wild-caught animals isolated from a small pond near Lake Orta, Italy. Populations were grown in sterile distilled water and fed with autoclaved and filter-sterilised organic lettuce extract. Prior to DNA extraction, animals were washed twice in sterile distilled water and starved overnight (approximately 16 hours) before being washed again with HyPure molecular-grade water. Genomic DNA from approximately 420 animals (260 derived from a single founding animal; the remainder derived from a subpopulation of approximately 10 wild-caught founders) was extracted using the DNeasy Blood & Tissue kit (Qiagen) following the standard protocol. DNA was extracted in batches and pooled to generate sufficient material. Paired-end data for *R*. *magnacalcarata* have been described previously [41]. Both *R*. *macrura* and *R*. *magnacalcarata* PE libraries are derived from multiple individual samples. For mate-pair library construction for both *R*. *macrura* and *R*. *magnacalcarata*, DNA was extracted from a single individual and subjected to WGA using the Repli-G Single Cell kit (Qiagen), following the manufacturer’s protocol. DNA concentration and quality were ascertained using a Qubit (Invitrogen) and a NanoDrop spectrophotometer (Thermo Scientific). Desiccation tolerance of *R*. *macrura* and *R*. *magnacalcarata* were tested using protocols as previously described [17,41] (see S3 Text for further details).

### Sequencing

For *A*. *ricciae*, a short-insert library with an insert size of 250 bp was prepared using Illumina Nextera reagents and sequenced (100 bases paired-end) on an Illumina HiSeq 2000 at the Eastern Sequence and Informatics Hub (Cambridge, UK). Two long-insert (mate-pair) libraries both with inserts of 3 kb were also sequenced (51 bases paired-end) at GATC Biotech (London, UK). In addition, a PacBio (Pacific Biosciences) long-read library with an insert of 10 kb was sequenced using 3 SMRT Cells on a PacBio RS II (The Genome Analysis Centre, Norwich, UK). An RNASeq library with an insert size of 250 bp was sequenced (150 bases paired-end) on an Illumina NextSeq500 at the Department of Biochemistry, University of Cambridge (Cambridge, UK). A short-insert library (500-bp insert) for *R*. *macrura* was prepared using Illumina TruSeq reagents at the Centre for Genomic Research (CGR) at the University of Liverpool (Liverpool, UK). Mate-pair libraries with 2-kb inserts were also prepared at CGR using Nextera reagents, and all libraries were sequenced (150 bases paired-end) over 3 lanes of an Illumina HiSeq4000 at CGR. Short-insert data for *R*. *magnacalcarata* have been described previously [41]. All raw data have been submitted to the Sequence Read Archive (SRA), an International Nucleotide Sequence Database Collaboration (INSDC), under the accession IDs ERR2135445–55 (S1 Table).

### Genome assembly

For *A*. *ricciae*, *R*. *macrura*, and *R*. *magnacalcarata* data, adapter sequences and low-quality bases were removed from Illumina data using Skewer v0.2.2 [107], and data quality was manually assessed using FastQC v0.11.5 [108]. Genome coverage was estimated by generating kmer distributions using BBMap ‘kmercountexact’ v36.02 [109], and library insert sizes—along with initial genome size estimates—were calculated using SGA ‘preqc’ [110]. Error correction of reads was performed using BBMap ‘tadpole’ (*k* = 31), discarding any pairs of reads containing unique kmers.

Contaminant reads derived from non–target organisms were filtered using BlobTools v0.9.19 [111]. Briefly, trimmed and error-corrected paired-end data were digitally normalised to approximately 100x using BBMap ‘bbnorm’ [109], and a preliminary draft assembly was generated using Velvet v1.2.10 [112], setting a kmer length of 75. Taxonomic annotations for all contigs were determined by comparing contigs against the NCBI nucleotide database (nt) and a custom database containing recently published whole-genome sequences of metazoans within the Spiralia (Lophotrochzoa) group (S4 Table) using BLAST ‘megablast’ (*E*-value ≤1 × 10^−25^) [113], and the UniRef90 database using Diamond ‘blastx’ [114]. Finally, read coverage for each contig was estimated by mapping non-normalised reads to each draft assembly using BWA ‘mem’ v0.7.12 [115]. Taxon-annotated GC coverage plots (‘blobplots’) [111,116] were generated using BlobTools (default parameters) and inspected manually. Putative contaminant sequences were identified as contigs showing atypical GC content, read coverage, and/or taxonomic classification. Given the a priori expectation that a substantial number of bdelloid genes may derive from nonmetazoan sources, we did not exclude any contigs based on taxonomy alone. Paired reads were excluded from further analysis only if both mapped to an identified contaminant contig or if one of the pair mapped to a contaminant while the other was unmapped. Additional rounds of filtering were performed if previously unassembled contaminant sequences became evident upon reassembly.

Filtered reads were assembled into contigs using the Platanus assembler v1.2.4 [42] with default parameters. Mate-pair libraries were filtered to remove contaminating FR-orientated reads (i.e., reads originating from short fragments) by excluding reads that mapped within ≤500 bases from the terminus of a contig. Contigs were scaffolded using SSPACE v3.0 [117], and undetermined bases were filled using GapFiller v1.10 [118]. The *A*. *ricciae* assembly was further scaffolded with the PacBio library using SSPACE-LongRead v1.1 [119]. RNASeq reads for *A*. *ricciae* were assembled de novo using Trinity v2.2.0 [120] (default parameters) and used for additional scaffolding with L_RNA_Scaffolder [121] and SCUBAT v2 [122]. An available transcriptome for *R*. *magnacalcarata* [41] was similarly utilised. A final round of assembly ‘polishing’ was performed using Redundans v0.12b [43], and scaffolds less than 200 bases in length were discarded. Assembly completeness was evaluated using the CEGMA v2.5 [44] and BUSCO v3.0.0 [45] gene sets, choosing the Eukaryota (*n* = 303) and Metazoa (*n* = 978) databases in the latter case and increasing the search limit to 8. Alternative assemblies were also generated using Velvet [112], SPAdes [123], and dipSPAdes [124] for comparison.

The reference assembly pipeline above was designed to maximise assembly contiguity but may lead to assembly collapse, the extent of which is undetermined a priori. Therefore, maximum haplotype assemblies were also generated for each species for comparison, defined as assemblies with minimal reduction due to assembly collapse. Maximum haplotype assemblies were generated using either dipSPAdes (default settings) or Platanus with the ‘bubble crush’ reduction parameter set to 0. Details of assembly parameters trialled are given in S1 Data. Collapsed and maximum haplotype (re)assemblies for *A*. *vaga* were also generated following the same procedures, using Illumina short-insert libraries (accession IDs SRR801084 and ERR321927) for contig building as well as mate-pair (accession ID ERR321928) and 454 data for scaffolding (see [28] for details).

### Gene prediction

Repetitive regions were masked prior to gene prediction. Repeats were modelled ab initio using RepeatModeler v1.0.5 [125]. Repeats arising from duplicated genes or recent gene family expansions (e.g., alpha-tubulin in *R*. *magnacalcarata* [126]) were removed from the custom repeat library by comparing each repeat library to the SwissProt database (BLASTX, *E*-value ≤1 × 10^−5^) and retaining only those sequences with descriptions for known repeat elements. The filtered RepeatModeler library was merged with known Rotifera repeats from Repbase v22.02 [127] (accessed using the command ‘queryRepeatDatabase.pl -species ‘rotifera’) and compared to each assembly using RepeatMasker v4.0.7 [128]. Low-complexity regions and simple repeats were additionally soft-masked.

Gene prediction was then performed using BRAKER v1.9 [47] where RNASeq data was available (*A*. *ricciae* and *R*. *magnacalcarata*). Briefly, RNASeq reads were aligned to the masked assembly using STAR, specifying the ‘twoPassMode Basic’ parameter to improve splice junction annotation. The resultant alignment BAM file was then input to the BRAKER pipeline with default settings. For *R*. *macrura*, an initial set of gene models was constructed using MAKER v3.00 [48], using evidence from SNAP [129] and GeneMark-ES v4.3 [130]. MAKER-derived gene models were then passed to Augustus v3.2.1 [49] for final refinement. Transfer and ribosomal RNA genes were predicted using tRNAscan-SE v1.3.1 [131] and RNAmmer [132], respectively (S9 Data). The *A*. *vaga* 2013 assembly (GCA_000513175.1) was also reannotated for consistency with these results, using both approaches outlined above (in conjunction with RNASeq library accession ERR260376).

To test if CDSs had been inadvertently missed during gene prediction, we compared proteins to the source nucleotide sequences from which they had been predicted using TBLASTN (*E*-value ≤1 × 10^−20^). Matches to existing gene models were discounted by removing alignments that showed any overlap with gene regions (BEDtools ‘intersect’ [133] with the ‘-v’ option), leaving only hits to regions of the genome that had not already been annotated as a gene.

### Collinearity analyses

Syntenic regions within and between genomes were identified using MCScanX [50], calling collinear ‘blocks’ regions with at least 5 homologous genes and fewer than 10 ‘gaps’ (i.e., missing genes). Rates of synonymous (*K*_S_) and nonsynonymous (*K*_A_) substitution between pairs of collinear genes were estimated by aligning proteins with Clustal Omega [134] and back-translating to nucleotides before calculating *K*_A_ and *K*_S_ values using BioPerl [135]. The collinearity of each block was calculated by dividing the number of collinear genes in a block by the total number of genes in the same region [28]. We also counted the number of collinearity breakpoints between adjacent homologous blocks across each genome, defining a breakpoint as an occurrence in which homologous blocks cannot be aligned without rearrangement. Collinearity plots were generated using the Circos software [136] in conjunction with the circosviz.pl program from the mmgenome toolkit [137]. Collinearity analysis scripts are available at https://github.com/reubwn/collinearity.

### Orthologous clustering and SNP finding

Orthologous relationships among proteins from the same set of protostomes as above were inferred using OrthoFinder v1.1.4 [138] with default settings. All genomic, GFF, and protein sequence datasets were downloaded from NCBI GenBank no later than May 2017. For SNP finding, data were mapped using Bowtie2 v2.2.6 [139] with the ‘--very-sensitive’ preset to minimise mismapped reads, and SNPs and indels were called using Platypus v0.8.1 [140], setting a minimum mapping quality of 30, a minimum base quality of 20, filter duplicates to 1, and a minimum read depth to approximately 25% of the average coverage of each individual library. VCF manipulation and SNP statistics were calculated using VCFlib v1.0.0-rc1 [141]. For *A*. *vaga*, SNPs were called based on the Illumina dataset ERR321927 mapped to the published genome sequence [28]. For *R*. *macrura* and *R*. *magnacalcarata*, SNPs were called based on WGA mate-pair libraries mapped as single-end because paired-end data for these samples were composed of multiple nonclonal lineages.

### HGT analyses

We assessed the extent of horizontal transfer into bdelloid genomes using a combination of sequence comparison and phylogenetics-based approaches and applied the same tests to a set of 13 publicly available proteomes from species across the Protostomia (S4 Table). Protein sequences were first compared to the UniRef90 database [142] (downloaded November 29, 2016) using Diamond ‘blastp’ [114] (*E*-value ≤1 × 10^−5^; maximum target sequences = 500). To avoid potential bias from bdelloid sequences already submitted to GenBank, all hits to the phylum Rotifera (NCBI taxonomy ID 10190) were omitted from further analysis. For each query, 2 HGT metrics were then calculated: (1) HGT Index (*h*_*U*_ [27]), defined as *B*_OUT_ − *B*_IN_, where *B*_IN_ is the best (highest) Diamond bitscore from comparisons to ‘ingroup’ taxa and *B*_OUT_ is the corresponding score for hits to ‘outgroup’ taxa; and (2) Consensus Hit Support (CHS), defined as the proportion of all hits that support a given query’s ingroup/outgroup classification, itself inferred from the highest sum of bitscores to ingroup or outgroup across all hits [94]. The CHS score therefore takes into account the taxonomic distribution of all hits for each query and militates against misclassifications based on *h*_*U*_ scores alone. We defined the ingroup as ‘Metazoa’ and the outgroup as ‘non-Metazoa’ and marked all proteins with an *h*_*U*_ ≥30 and CHS_OUT_ ≥90% as putative HGT_C_. We then looked at the distribution of all HGT_C_ across the genome and discarded any candidate found on a scaffold encoding ≥95% of genes of putative foreign origin (i.e., ‘HGT-heavy’ scaffolds that may be derived from contaminant sequences that were not removed during assembly). For each HGT_C_, physical linkage (i.e., presence on the same scaffold) to a gene with good evidence for metazoan origin (*h*_*U*_ ≤0, CHS_IN_ ≥90%) and the number of predicted introns were also recorded. Finally, phylogenetic support for HGT was then assessed: for each HGT_C_, the sequences of 15 metazoan and 15 nonmetazoan UniRef90 hits (when present) were extracted and aligned using MAFFT v7.309 [143] with default parameters, and a maximum likelihood phylogeny was constructed using IQ-TREE v1.5.3 [144], specifying automatic model selection and 1,000 ultrafast bootstrap replicates. The functionality of GNU Parallel [145] was used to compute multiple trees simultaneously, and clusters with fewer than 4 taxa were not analysed. Branching patterns of resultant trees were then assessed using a custom script written in R v3.3.1 [146], utilising functions from the ‘ape’ v4.1 package [147]. HGT analysis scripts are available at https://github.com/reubwn/hgt.

### TE analyses

The abundance of known TEs was assessed for the same set of protostomes using RepeatMasker, except using a Repbase (v22.02) repeat library specific to the Metazoa (i.e., ‘queryRepeatDatabase.pl -species ‘metazoa”). Custom species-specific repeat libraries (e.g., using RepeatModeler) were not generated for this analysis; only known repeats from Repbase were compared. The total span of LINEs/SINEs, LTR elements, DNA elements, and simple repeats relative to the assembly span for each species was then computed from the RepeatMasker results. We also calculated a genome density metric, defined as the number of protein-coding genes per Mb of haploid genome, i.e., accounting for variation in ploidy among species.

### Meiosis genes

The presence of meiosis- and other sex-related genes was assessed following the approach of Tekle et al. [101]. A total of 41 orthologous groups were downloaded from the OrthoMCL database (v5) (http://orthomcl.org/orthomcl/; accessed September 2017) (S8 Data). Searches were conducted using both TBLASTN (*E*-value ≤1 × 10^−5^) against the reference assemblies or HMMER3 (http://hmmer.org/) against the predicted protein sets, after alignment with Clustal Omega [134]. Presence was recorded if any query within each orthologous group showed a TBLASTN alignment with ≥50% identity over ≥50% query length and/or if HMMER reported an alignment above the default significance threshold. Multiple hits to the same location (caused by paralogy or hits to similar domains) were recorded if top hits overlapped among queries. The genomes and proteomes of *D*. *melanogaster* and *C*. *elegans* were also searched for comparison.

## Supporting information

S1 Data Assembly metrics for reference and maximum haplotype assemblies. (XLSX)

S2 Data Augustus GFF and protein FASTA files (filtered). (TGZ)

S3 Data Repeat content. (XLSX)

S4 Data MCScanX and collinearity analyses. (TGZ)

S5 Data SNP information. SNP, single-nucleotide polymorphism.
(XLSX)

S6 Data Platypus VCF files. (TGZ)

S7 Data HGT metrics. HGT, horizontal gene transfer.
(XLSX)

S8 Data Meiosis- and sex-related gene inventory. (XLSX)

S9 Data Transfer and ribosomal RNA gene annotation. (TGZ)

S1 Fig Kmer spectra for raw and filtered sequence data. (PDF)

S2 Fig Taxon-annotated GC coverage plots for initial and final assemblies. GC, guanine-cytosine.
(PDF)

S3 Fig Effect of bubble crush on Platanus assembly. (PDF)

S4 Fig Effect of Redundans reduction. (PDF)

S5 Fig Distribution of intergenic distances between genes. (PDF)

S6 Fig Distribution of intron length. (PDF)

S7 Fig MAF distributions for paired-end *Rotaria* sequence data. MAF, minor allele frequency.
(PDF)

S8 Fig Coverage profiles for reference and alternative assemblies for *A*. *vaga*. (PDF)

S9 Fig Coverage profiles for reference and alternative *A*. *ricciae* assemblies. (PDF)

S10 Fig Coverage profile and SNP density distribution for *A*. *ricciae* homologous genes. SNP, single-nucleotide polymorphism.
(PDF)

S11 Fig Genome size estimates based on kmer spectra. (PDF)

S12 Fig Orthologous clustering within bdelloid genomes. (PDF)

S13 Fig Evidence for ancient origin for most HGT_C_ genes in bdelloids. HGT_C_, horizontal gene transfer candidates.
(PDF)

S1 Table Data counts and accession numbers for sequence data used in this study. (PDF)

S2 Table MCScanX collinearity metrics within genomes. (PDF)

S3 Table Homologous and ohnologous *K*_A_ and *K*_S_. (PDF)

S4 Table Protostome species included in comparative analysis. (PDF)

S5 Table OrthoFinder clustering metrics. (PDF)

S6 Table OrthoFinder clustering metrics per species. (PDF)

S1 Text Signals of duplication and collapse in the *A*. *ricciae* sequence data. (PDF)

S2 Text Note on bdelloid genome size estimates. (PDF)

S3 Text Test of desiccation tolerance for *Rotaria* species. (PDF)

## Abbreviations

BUSCO: Benchmarking Universal Single-Copy Orthologs
CDS: coding sequence
CEGMA: Core Eukaryotic Gene Mapping Approach
CGR: Centre for Genomic Research
CHS: Consensus Hit Support
DSB: double-strand break
GC: guanine-cytosine
HGT: horizontal gene transfer
HGT_C_: HGT candidate
INSDC: International Nucleotide Sequence Database Collaboration
LINE: long interspersed element
LTR: long terminal repeat
MAF: minor allele frequency
RNASeq: RNA sequencing
SEG: single-exon gene
SINE: short interspersed element
SNP: single-nucleotide polymorphism
SRA: Sequence Read Archive
TE: transposable element
WGA: whole-genome amplification
