# Supplementary material for: Comparative genomics of bdelloid rotifers: Insights from desiccating and nondesiccating species
Source: PLoS Biol. 2018 Apr 24;16(4):e2004830. doi: 10.1371/journal.pbio.2004830 (PMC5916493; doi:10.1371/journal.pbio.2004830)
Supplement: S1 Fig — (PDF) [file pbio.2004830.s010.pdf]

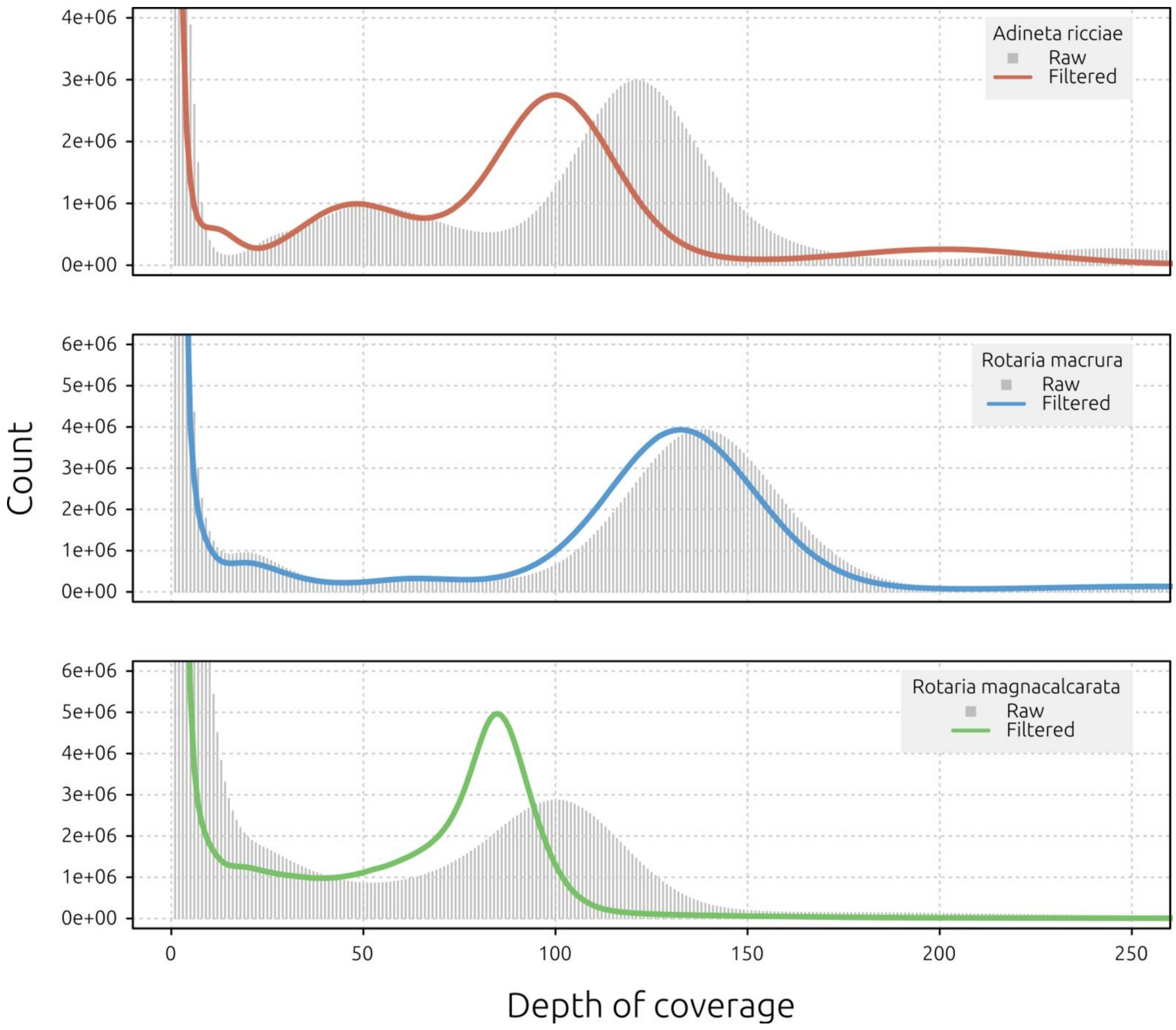

S1 Fig. Kmer spectra for raw and filtered sequence data. Distributions show kmer depths (*k* = 31) in raw (grey bars) and filtered (coloured lines) sequence data. Arrows indicate potential secondary and tertiary coverage peaks. The large number of low coverage kmers in the *Rotaria* datasets (particularly *R. magnacalcarata*) indicate substantial levels of polymorphism, most likely due to the sampling approach.
