# Supplementary material for: Comparative genomics of bdelloid rotifers: Insights from desiccating and nondesiccating species
Source: PLoS Biol. 2018 Apr 24;16(4):e2004830. doi: 10.1371/journal.pbio.2004830 (PMC5916493; doi:10.1371/journal.pbio.2004830)
Supplement: S2 Fig — GC, guanine-cytosine. (PDF) [file pbio.2004830.s011.pdf]

(A)

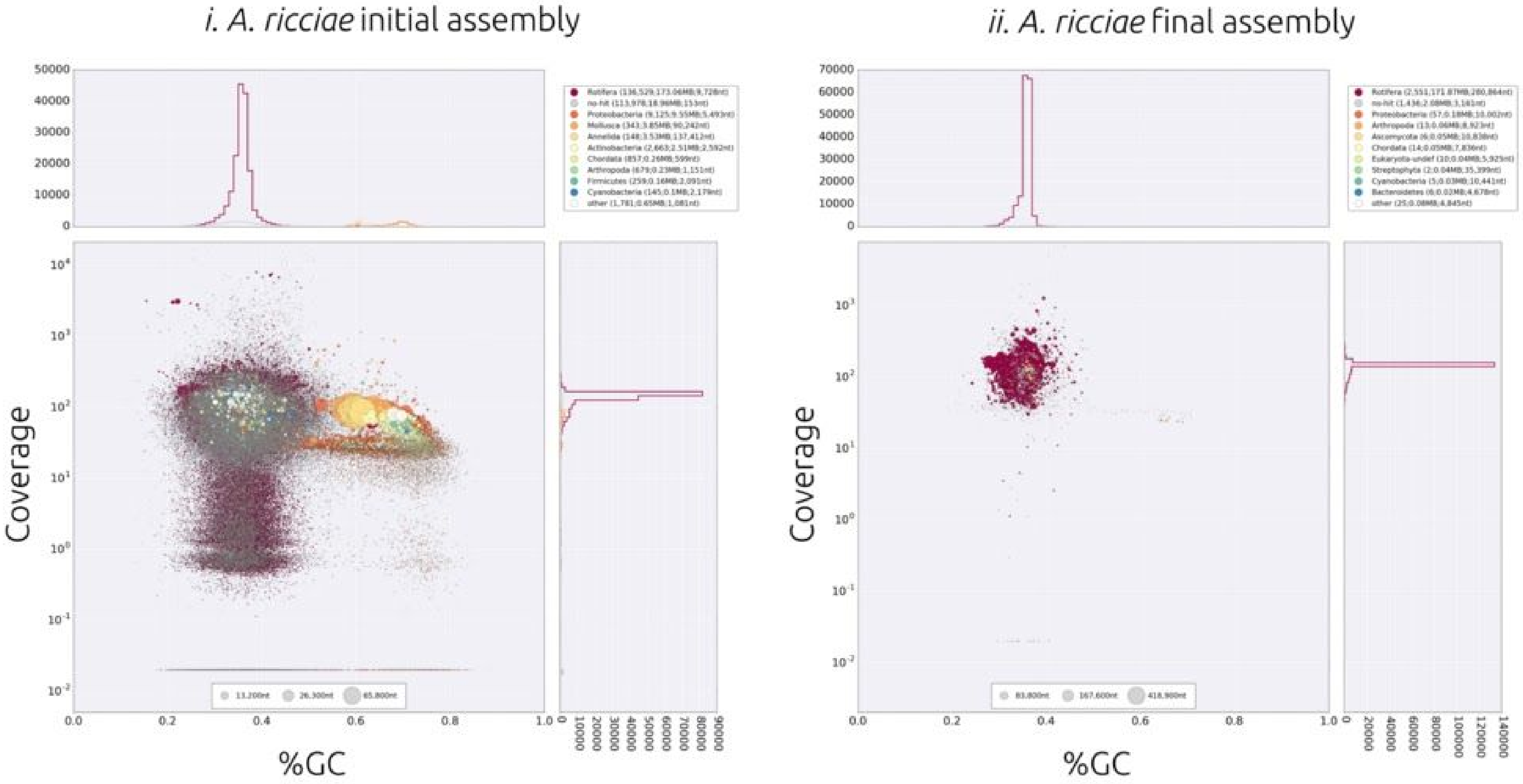
i. A. ricciae initial assembly
ii. A. ricciae final assembly
Coverage
%GC
Coverage
%GC

(B)

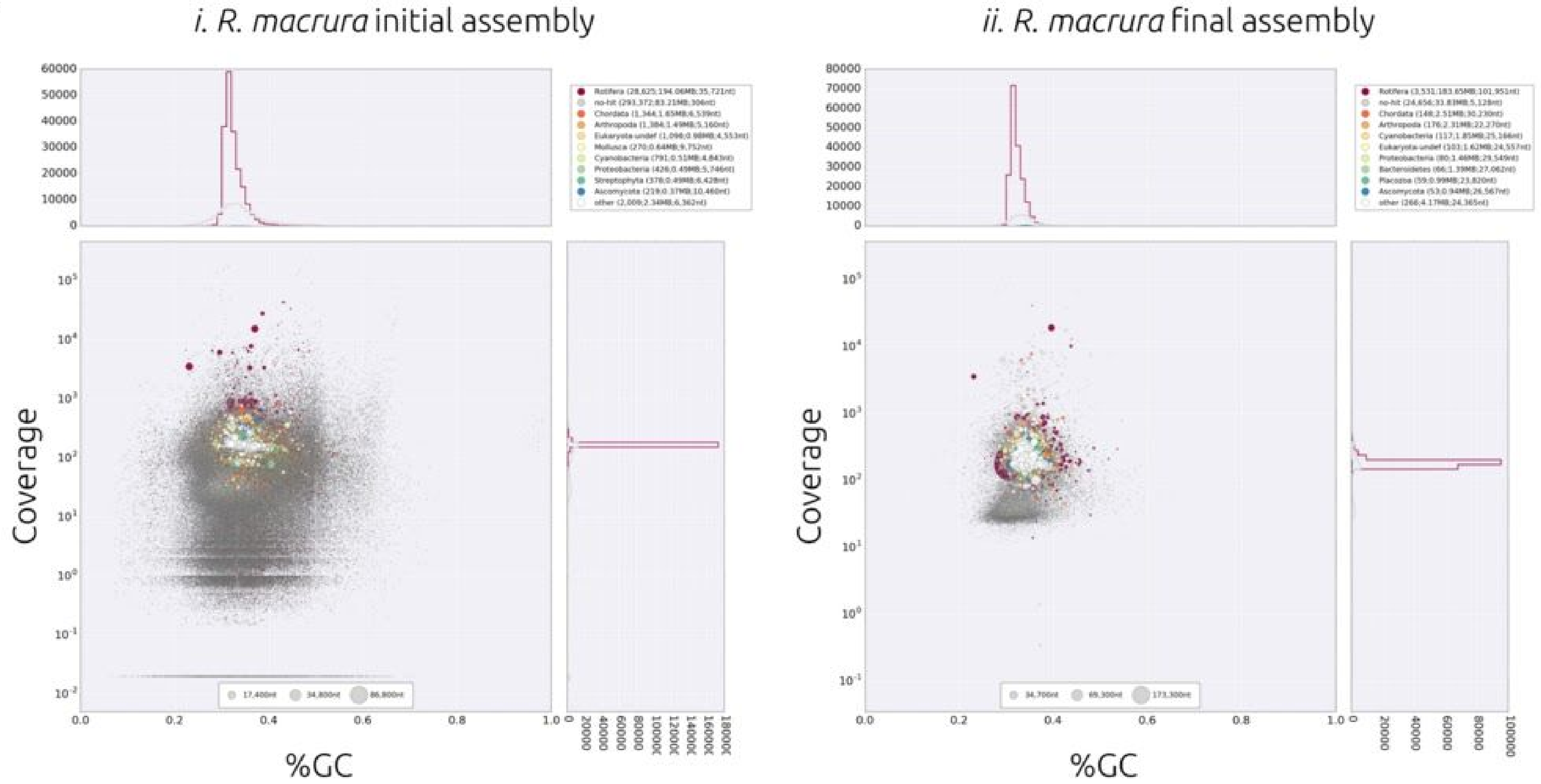

i. R. macrura initial assembly
ii. R. macrura final assembly
Coverage
%GC
Rotifera
no-hit
Chordata
Arthropoda
Eukaryota-undef
Mollusca
Cyanobacteria
Proteobacteria
Streptophyta
Ascomycota
Bacteroidetes
Placozoa
other

(C)

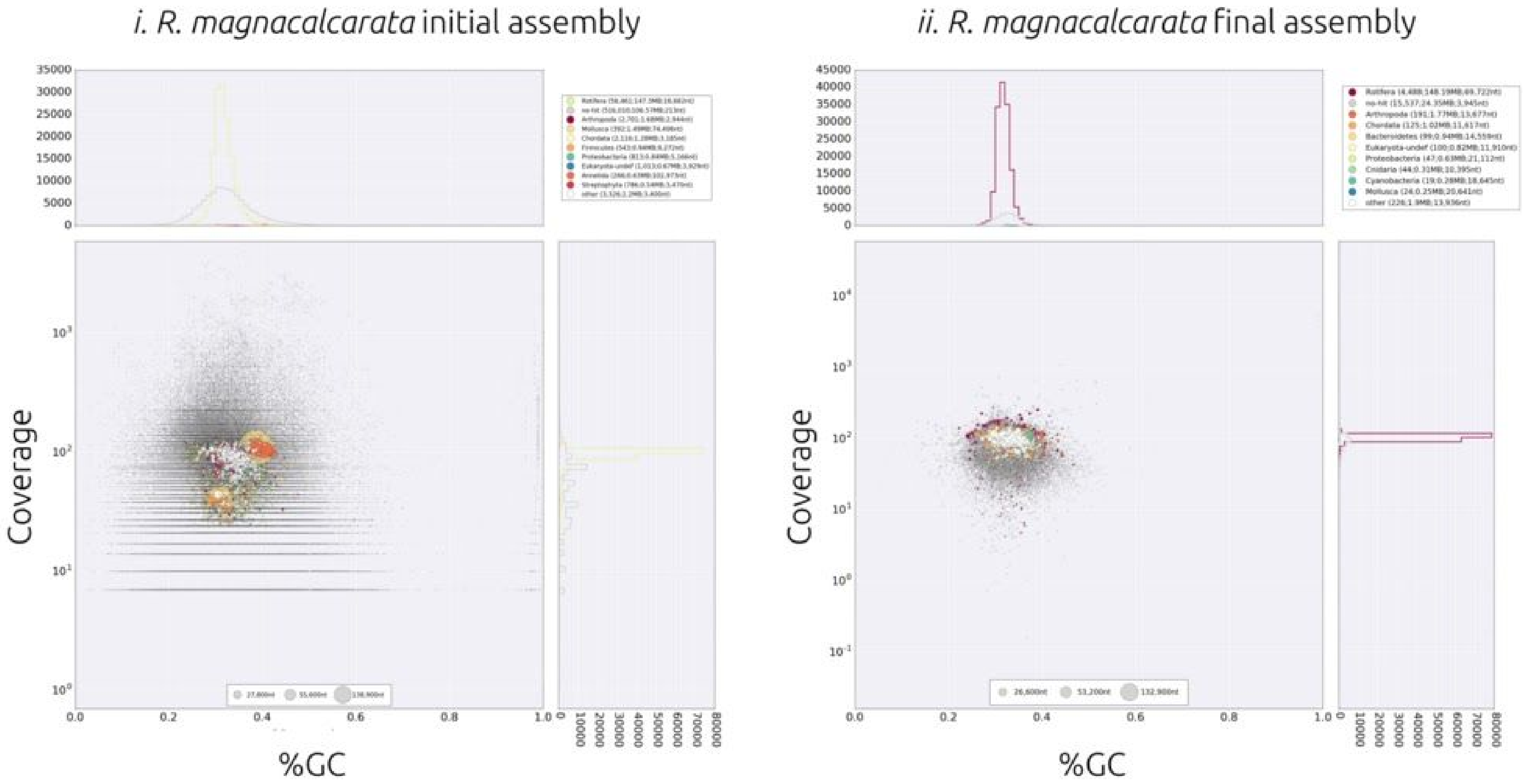

S2 Fig. Taxon-annotated GC-Coverage plots for initial and final assemblies. Initial draft assemblies for each species are shown in (i), final reference assemblies in (ii) for (A) *A. ricciae*, (B) *R. macrura* and (C) *R. magnacalcarata*. In each plot, circles represent assembly scaffolds with diameter proportional to the sequence length, positioned by %GC (*X*-axis) and average read coverage (*Y*-axis), and coloured by putative taxon of origin (see legends). Histograms in upper and right panels represent the total span of contigs/scaffolds in a given %GC or coverage bin.
