# Supplementary material for: Comparative genomics of bdelloid rotifers: Insights from desiccating and nondesiccating species
Source: PLoS Biol. 2018 Apr 24;16(4):e2004830. doi: 10.1371/journal.pbio.2004830 (PMC5916493; doi:10.1371/journal.pbio.2004830)
Supplement: S3 Fig — (PDF) [file pbio.2004830.s012.pdf]

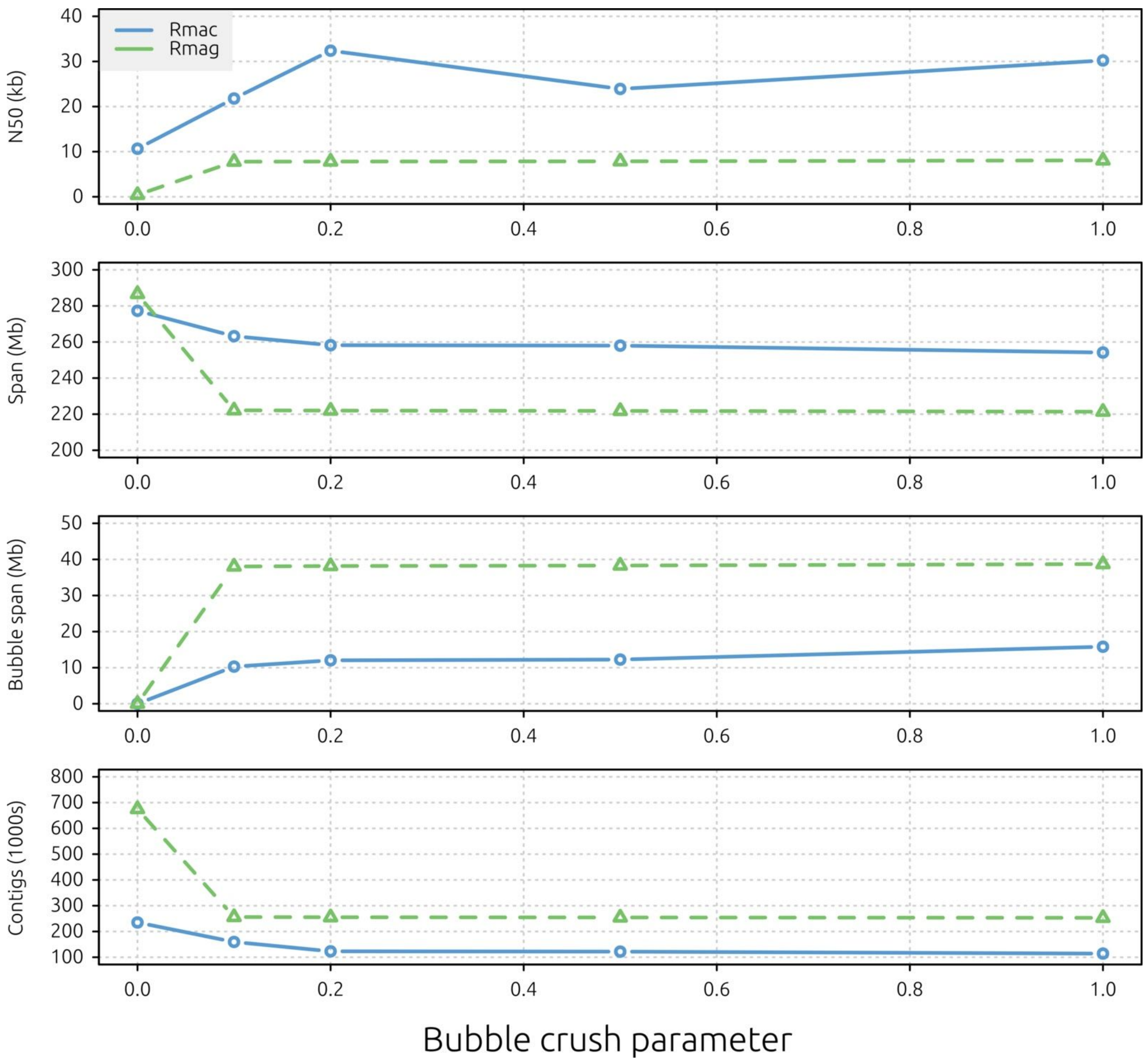

S3 Fig. Effect of bubble crush on Platanus assembly. Specifying the removal of polymorphic homologous regions, as identified by Platanus, results in substantial improvements to assembly contiguity by removing a large number of short contigs. Assembly improvements plateau at -u ≥ 0.2. "Bubble span" (third panel) indicates the total length of removed sequences. Values are based on assembly statistics for contigs ≤ 200 bp length.
