# Supplementary material for: Comparative genomics of bdelloid rotifers: Insights from desiccating and nondesiccating species
Source: PLoS Biol. 2018 Apr 24;16(4):e2004830. doi: 10.1371/journal.pbio.2004830 (PMC5916493; doi:10.1371/journal.pbio.2004830)
Supplement: S4 Fig — (PDF) [file pbio.2004830.s013.pdf]

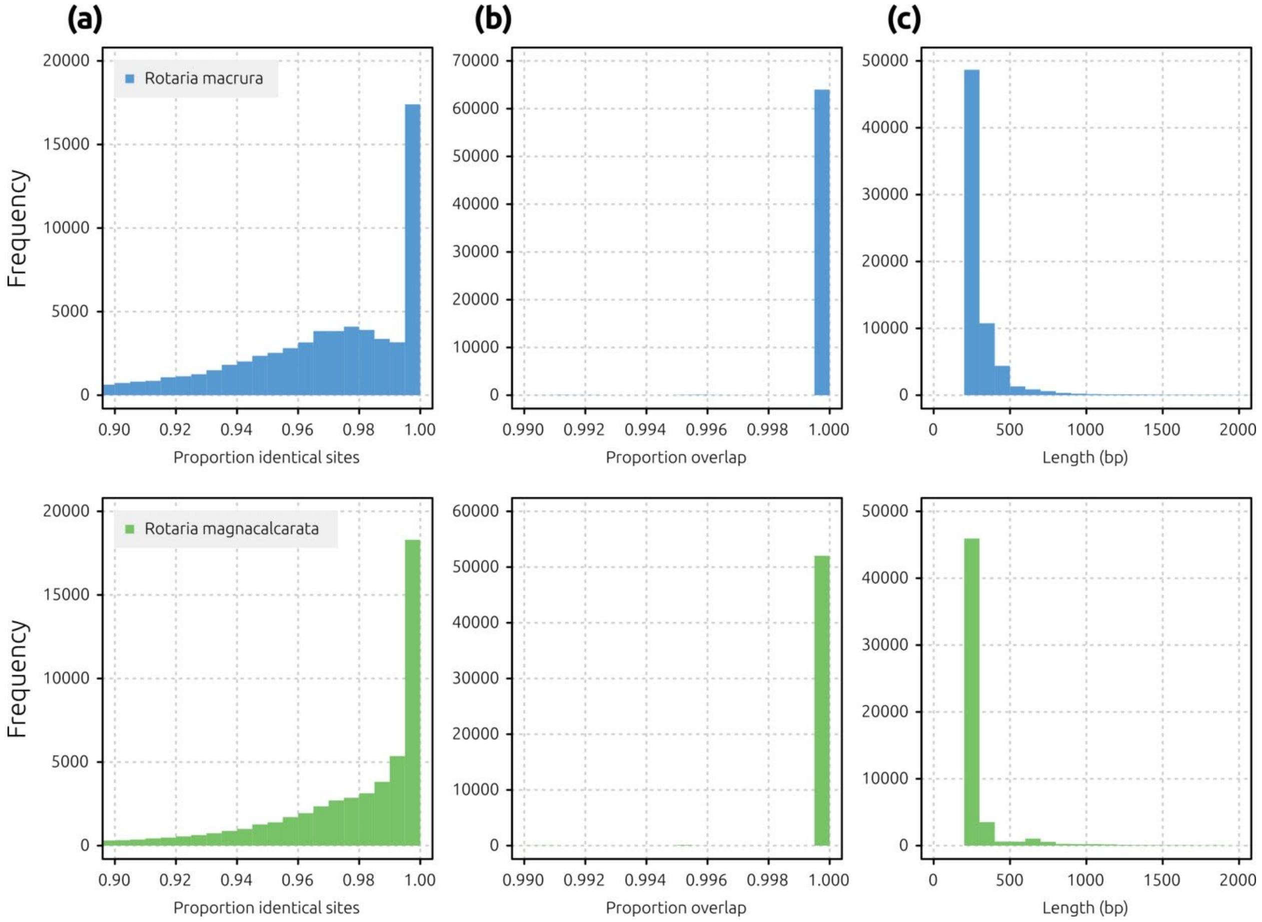

S4 Fig. Effect of Redundans reduction. (A) Identity distribution of discarded sequences to target sequences. (B) Proportion of overlap between discarded and target sequences. (C) Length distribution of discarded sequences.
