# Supplementary material for: Comparative genomics of bdelloid rotifers: Insights from desiccating and nondesiccating species
Source: PLoS Biol. 2018 Apr 24;16(4):e2004830. doi: 10.1371/journal.pbio.2004830 (PMC5916493; doi:10.1371/journal.pbio.2004830)
Supplement: S5 Fig — (PDF) [file pbio.2004830.s014.pdf]

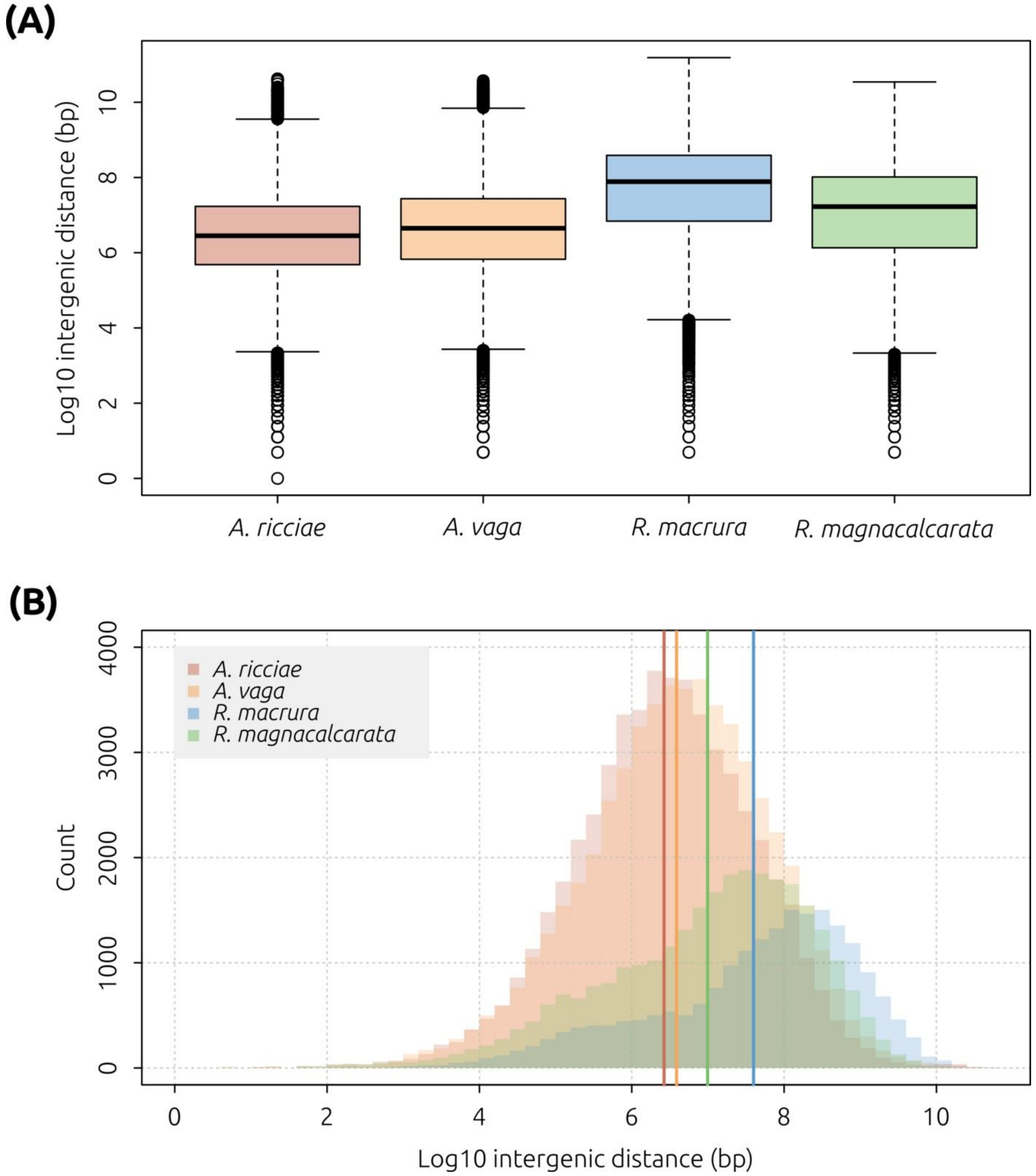

S5 Fig. Distribution of intergenic distances between genes. (A) Box-and-whisker plots showing interquartile range for intergenic distances between genes predicted on the same scaffolds in *A. ricciae* (red), *A. vaga* (orange), *R. macrura* (blue) and *R. magnacalcarata* (green). Median values are indicated by

a thick horizontal bar within boxes. Note $\log_{10}$ scale on *Y*-axis. (B) Overlayed distributions of intergenic distances for all species. Vertical lines indicate mean distances. Note $\log_{10}$ scale on *X*-axis.
