# Supplementary material for: Comparative genomics of bdelloid rotifers: Insights from desiccating and nondesiccating species
Source: PLoS Biol. 2018 Apr 24;16(4):e2004830. doi: 10.1371/journal.pbio.2004830 (PMC5916493; doi:10.1371/journal.pbio.2004830)
Supplement: S6 Fig — (PDF) [file pbio.2004830.s015.pdf]

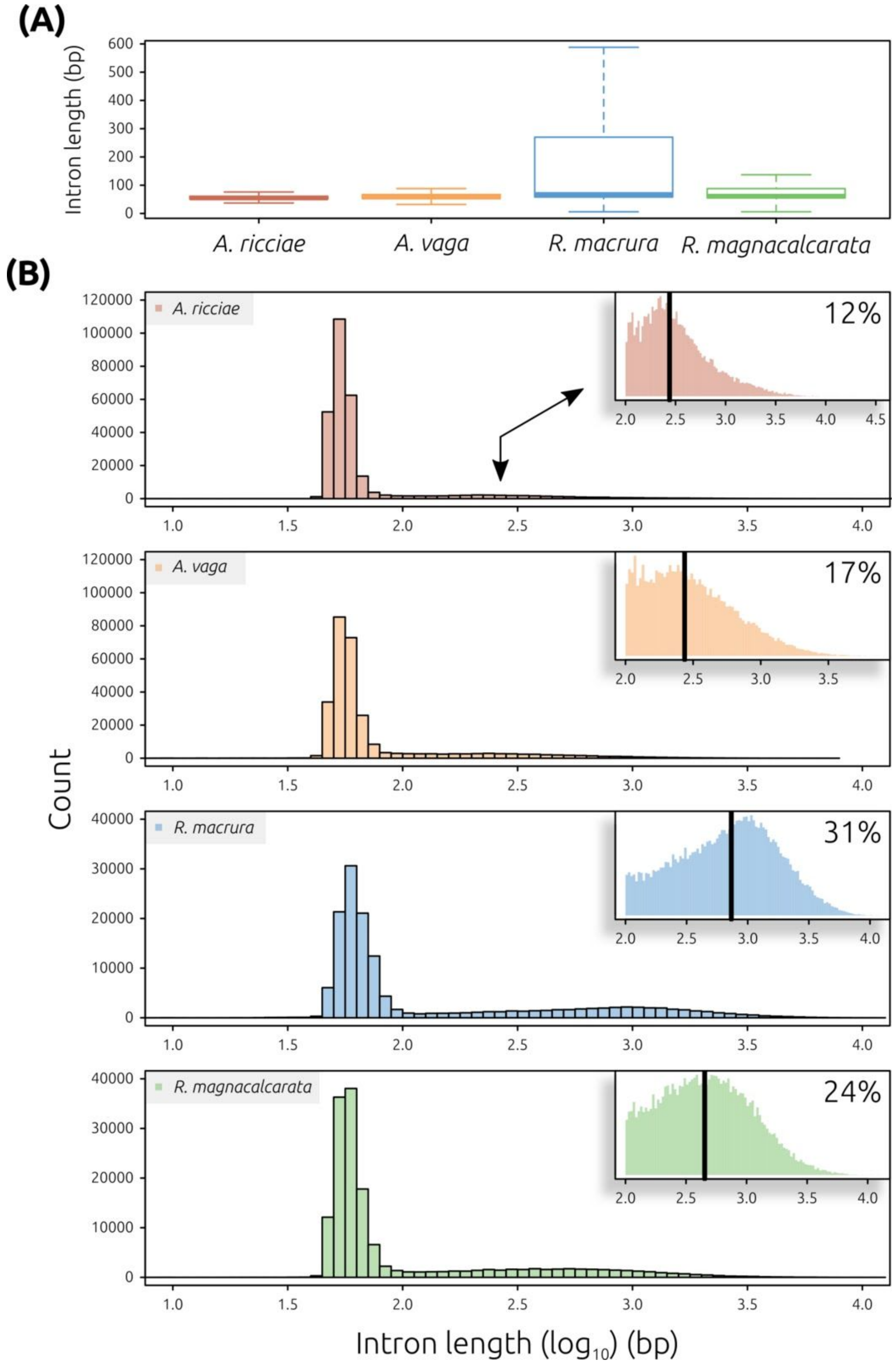

S6 Fig. Distribution of intron length. (A) Box-and-whisker plots showing interquartile range for all intron lengths in predicted genes from *A. ricciae* (red), *A. vaga* (orange), *R. macrura* (blue) and *R. magnacalcarata* (green). Median values are indicated by a thick horizontal bar within boxes. (B)

Distributions of intron size showing that most introns lie in the range 30–100 bp length, but a substantial minority are distributed around a much larger mean (inserts within each panel show distributions for introns > 100 bp length for detail, median values indicated with a black vertical bar). Note log_{10} scale on *X*-axis. The longer average introns in *R. macrura* are driven primarily by an upwards shift in the length of longer introns relative to the other species.
