# Supplementary material for: Comparative genomics of bdelloid rotifers: Insights from desiccating and nondesiccating species
Source: PLoS Biol. 2018 Apr 24;16(4):e2004830. doi: 10.1371/journal.pbio.2004830 (PMC5916493; doi:10.1371/journal.pbio.2004830)
Supplement: S7 Fig — MAF, minor allele frequency. (PDF) [file pbio.2004830.s016.pdf]

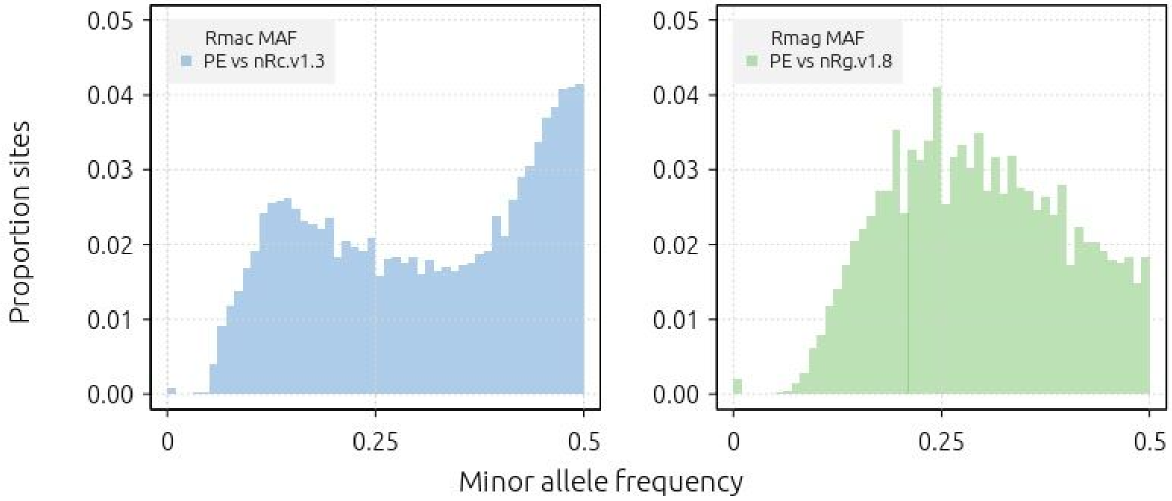

S7 Fig. MAF distributions for paired-end *Rotaria* sequence data. A high proportion of low frequency alleles is evident in both mappings, likely caused by population structure in the polyclonal samples (multiple wild-caught individuals) from which these data are derived—the mode at 0.5 is clearer in *R. macrura* as this sample consisted of fewer clones.
