# Supplementary material for: Comparative genomics of bdelloid rotifers: Insights from desiccating and nondesiccating species
Source: PLoS Biol. 2018 Apr 24;16(4):e2004830. doi: 10.1371/journal.pbio.2004830 (PMC5916493; doi:10.1371/journal.pbio.2004830)
Supplement: S8 Fig — (PDF) [file pbio.2004830.s017.pdf]

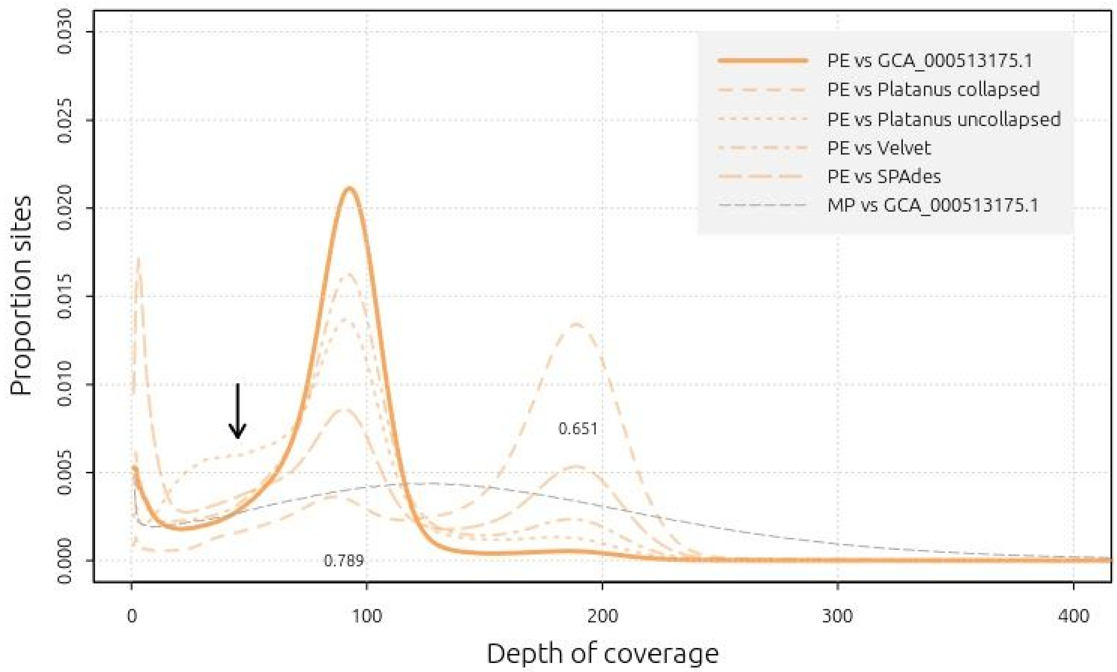

S8 Fig. Coverage profiles for reference and alternative assemblies for *A. vaga*. Read coverage distributions for data mapped to the *A. vaga* reference genome GCA_000513175.1 (the "2013" assembly) is bimodal, showing a major peak (78.9% sites) centered around ~90x and only a minor peak at ~180x. An uncollapsed alternative assembly shows a similar pattern but with the addition of a third coverage peak at approximately 40x (indicated with an arrow). When divergent homologous regions are removed, the majority of sites (65.1%) show coverage around 180x. Coverage determined from mapping PE library accession ERR321927 (Illumina HiSeq2000, 2 x100bp PE). Values under curves indicate proportion of sites under 50–130x (reference assembly, mode at 90x) and 150–230x (Platanus collapsed assembly, mode at 180x). Only coverage values ≥ 0 are plotted. Mate-pair (accession ERR321928) coverage is also shown.
