# Supplementary material for: Comparative genomics of bdelloid rotifers: Insights from desiccating and nondesiccating species
Source: PLoS Biol. 2018 Apr 24;16(4):e2004830. doi: 10.1371/journal.pbio.2004830 (PMC5916493; doi:10.1371/journal.pbio.2004830)
Supplement: S9 Fig — (PDF) [file pbio.2004830.s018.pdf]

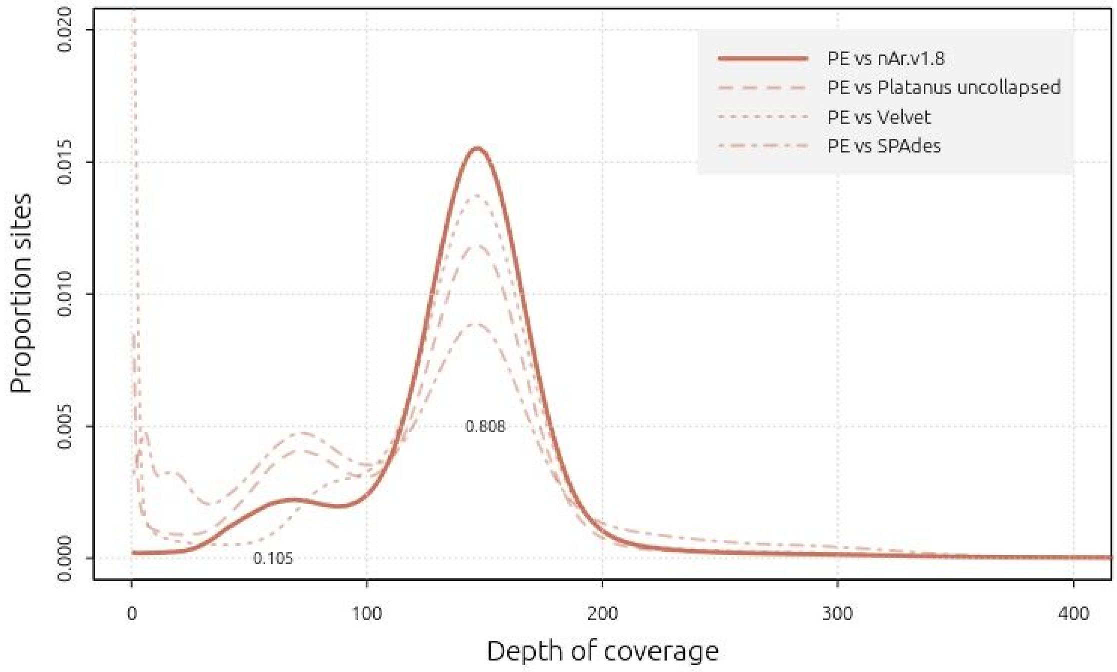

S9 Fig. Coverage profiles for reference and alternative *A. ricciae* assemblies. Paired-end data mapped to both the reference assembly (nAr.v1.8) and alternative assemblies built using Platanus (with polymorphism collapse disabled), Velvet and SPAdes all show similar multimodal coverage distributions, but in this case the majority of sites represented under the higher of the two major coverage peaks. The coverage profile for the reference genome has modes at approximately 75x (10.5% sites, area under curve from 30 to 90x) and again at 150x (80.8% sites, area under curve from 100 to 200x). Only coverage values ≥ 0 are plotted.
