# Supplementary material for: Comparative genomics of bdelloid rotifers: Insights from desiccating and nondesiccating species
Source: PLoS Biol. 2018 Apr 24;16(4):e2004830. doi: 10.1371/journal.pbio.2004830 (PMC5916493; doi:10.1371/journal.pbio.2004830)
Supplement: S10 Fig — SNP, single-nucleotide polymorphism. (PDF) [file pbio.2004830.s019.pdf]

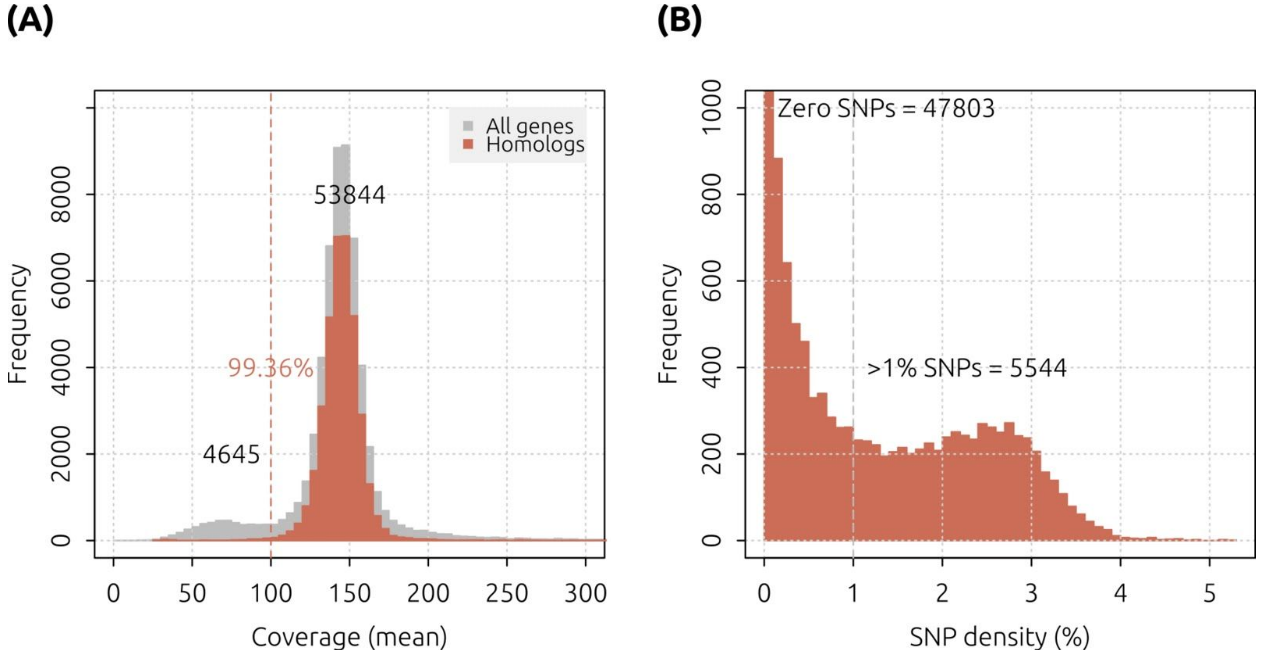

S10 Fig. Coverage profile and SNP density distribution for *A. ricciae* homologous genes. (A) Distribution of read coverage for all genes (grey bars) versus those with a homologous partner separately assembled elsewhere in the genome (red bars, inferred from MCScanX analysis), showing that virtually all (~99%) homologous genes are covered at 150x. The nature of those genes at the lower coverage value (75x) is yet to be determined. (B) Distribution of SNP density across all genes (defined as #SNPs per gene, normalised by gene length), showing high heterogeneity in SNP density across the genome: the majority of genes have zero detected SNPs (47,803, ~82%), and only relatively few genes (5,544, ~9%) show ≥ 1% SNP density.
