# Supplementary material for: Comparative genomics of bdelloid rotifers: Insights from desiccating and nondesiccating species
Source: PLoS Biol. 2018 Apr 24;16(4):e2004830. doi: 10.1371/journal.pbio.2004830 (PMC5916493; doi:10.1371/journal.pbio.2004830)
Supplement: S11 Fig — (PDF) [file pbio.2004830.s020.pdf]

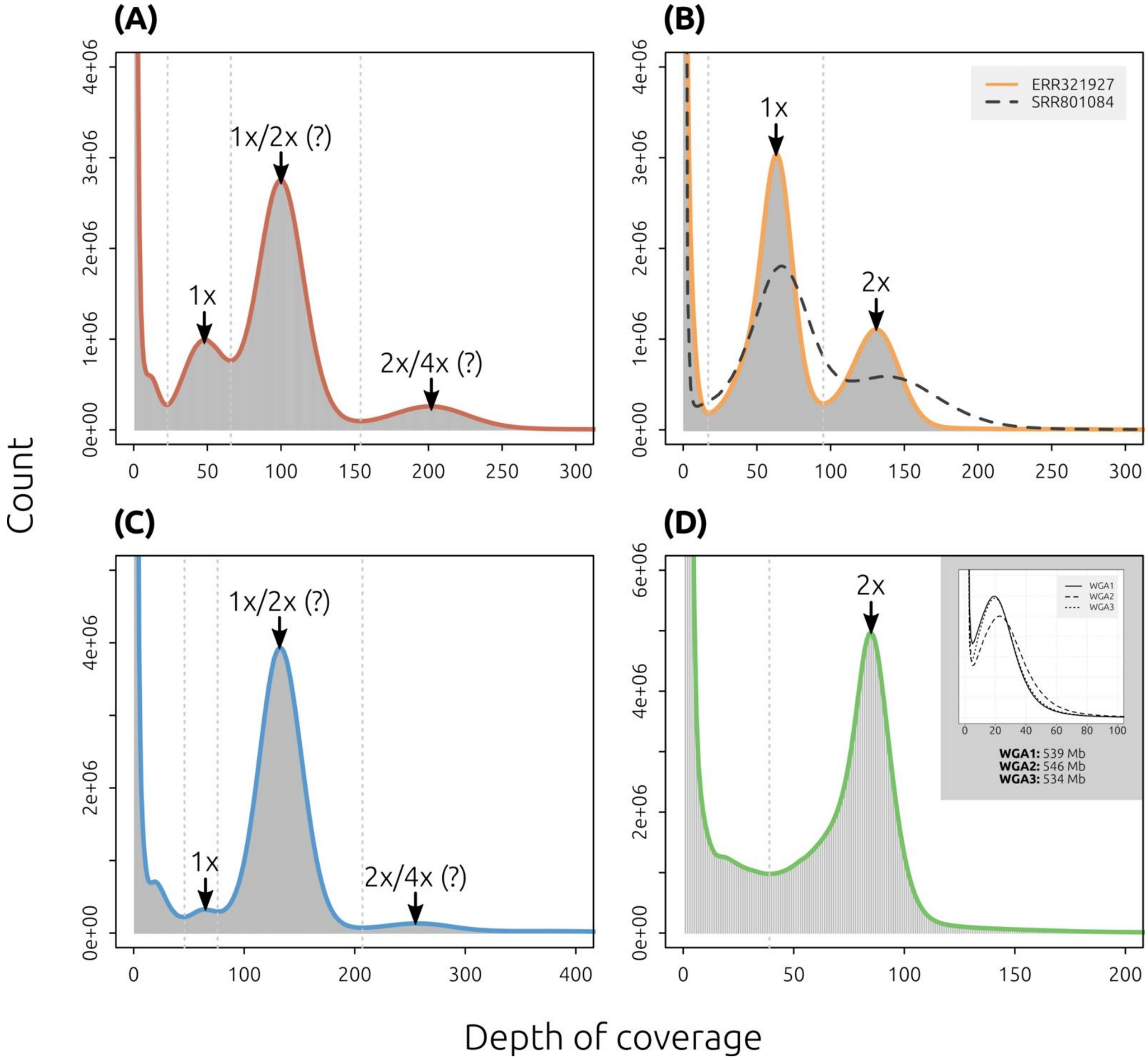

S11 Fig. Genome size estimates based on kmer spectra. Genome size can be approximated from kmer spectra by taking the product of the area under the curve (AUC) multiplied by the corresponding inferred copy number, summed across all peaks. However, this method is dependent on the correct assignation of observed peaks to copy number variation in the genome, which may be non-trivial in the case of these data, where multiple explanations are possible for observed peaks. Note this approach does not account for repeats at very high copy number (e.g., TEs). Spectra for *A. ricciae* (red), *A. vaga* (orange), *R. macrura* (blue) and *R. magnacalcarata* (green) are based on trimmed short-insert sequence data (**Table S1**) and were constructed using BBMap "kmercountexact" (k = 31). (A) *A. ricciae*. Assuming that the peak at 100x represents kmers in double copy as a result of

endoduplication in somatic nuclei and the peak at 200x is the result of homologous collapse, total genome size is estimated as 185.9 Mb. (B) *A. vaga*. Based on library ERR321927, assuming that the peak at 63x represents single copy kmers and the peak at 131x represents double copy kmers as a result of homologous collapse, total genome size is estimated as 191.4 Mb. Based on library SRR801084 (dashed line), total genome size is estimated as 196.2 Mb. (C) *R. macrura*. Assuming that the major peak at 132x represents double copy kmers as a result of homologous collapse, total genome size is estimated as 540.2 Mb. Note that the multimodality of this distribution bears some similarity to *A. ricciae*, however, and may point to a similar mechanism underlying both datasets. (D) *R. magnacalcarata*. Assuming the peak at 85x represents double copy kmers as a result of homologous collapse, total genome size is estimated as 310 Mb. This may be an underestimate due to the large proportion of kmers at lower coverage values caused by population-level polymorphisms (from non-clonal sample). Estimates from three independent whole-genome amplified (single individual) datasets suggest an increased total genome size of ~530–550 Mb (inset).
