# Supplementary material for: Comparative genomics of bdelloid rotifers: Insights from desiccating and nondesiccating species
Source: PLoS Biol. 2018 Apr 24;16(4):e2004830. doi: 10.1371/journal.pbio.2004830 (PMC5916493; doi:10.1371/journal.pbio.2004830)
Supplement: S12 Fig — (PDF) [file pbio.2004830.s021.pdf]

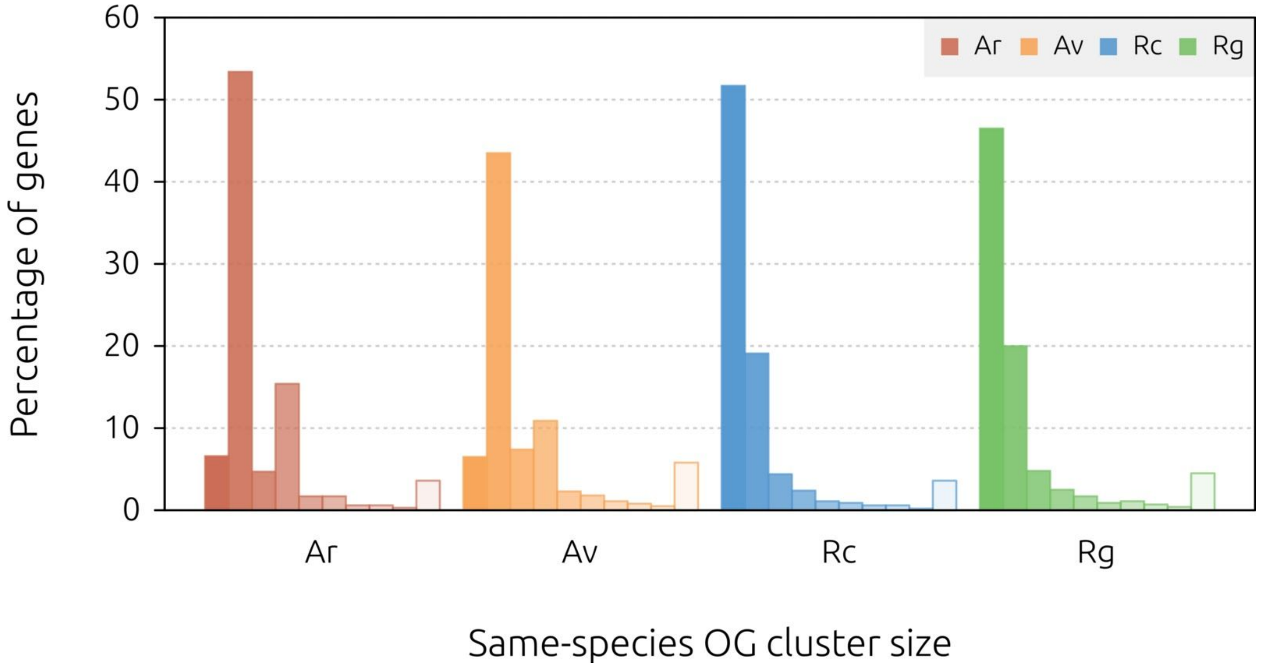

S12 Fig. Orthologous clustering within bdelloid genomes. Bars show the distribution of same-species co-orthologous cluster size, expressed as a proportion of each species genome (total number of CDS). Membership size ranges from one (i.e., singletons, left-hand bar) to 10+ (i.e., 10 co-orthologs from the same species, or more; right-hand bar).
