# Supplementary material for: Comparative genomics of bdelloid rotifers: Insights from desiccating and nondesiccating species
Source: PLoS Biol. 2018 Apr 24;16(4):e2004830. doi: 10.1371/journal.pbio.2004830 (PMC5916493; doi:10.1371/journal.pbio.2004830)
Supplement: S13 Fig — HGTC, horizontal gene transfer candidates. (PDF) [file pbio.2004830.s022.pdf]

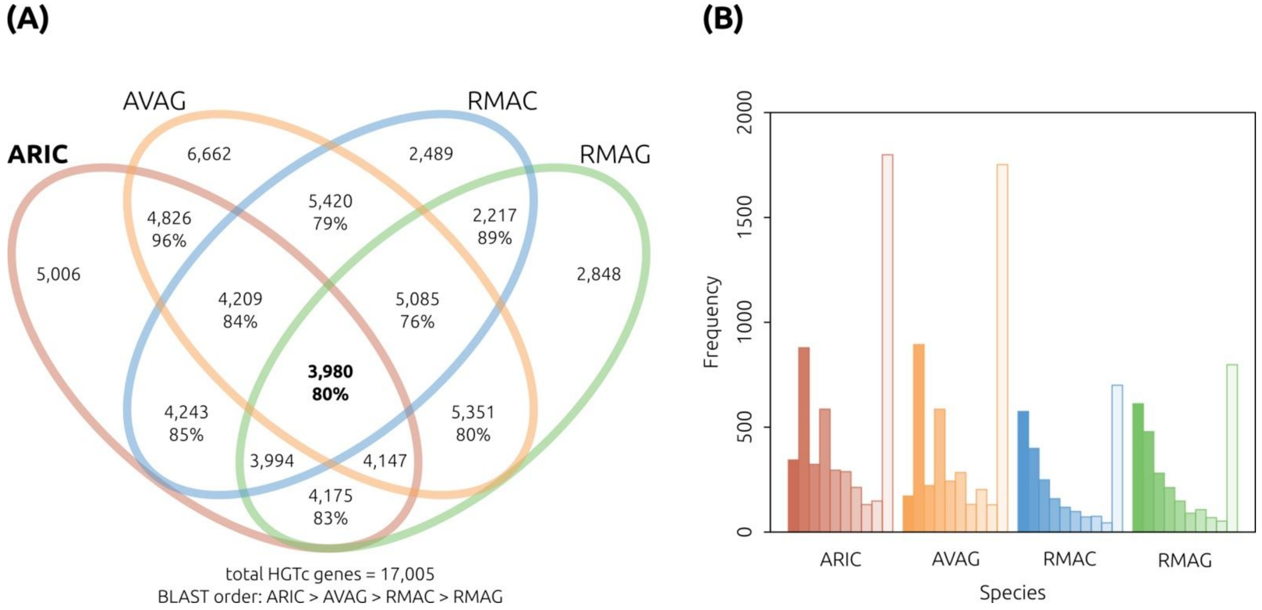

S13 Fig. Evidence for ancient origin for most HGT$_C$ genes in bdelloids. (A). Sequential BLAST analysis of HGT$_C$ genes. Values within overlapping segments represent the number/proportion HGT$_C$ present in each set, based on TBLASTN analysis (*E*-value $\leq$ 1e−5, accepting hits showing $\geq$ 60% query coverage). All values are relative, based on the order *A. ricciae* (ARIC) > *A. vaga* (AVAG) > *R. macrura* (RMAC) > *R. magnacalcarata* (RMAG). Thus, 3,980 (80%) of *A. ricciae* HGT$_C$ genes are found in all four species; 5,085 (76%) of *A. vaga* HGT$_C$ genes are found in *R. macrura* and *R. magnacalcarata*, etc. (B) OG membership distribution for HGT$_C$ genes. For each species, bars from left to right represent the number of co-orthologous genes for HGT$_C$ genes (based on BLASTP hits showing $\geq$ 60% query coverage as above). The shape of each distribution reflects that of all genes (**S12 Fig**), except many HGT$_C$ genes show 10+ hits to other proteins, suggesting that some HGT$_C$ genes may have undergone substantial duplication after domestication.
