# Supplementary material for: Comparative genomics of bdelloid rotifers: Insights from desiccating and nondesiccating species
Source: PLoS Biol. 2018 Apr 24;16(4):e2004830. doi: 10.1371/journal.pbio.2004830 (PMC5916493; doi:10.1371/journal.pbio.2004830)
Supplement: S1 Table — (PDF) [file pbio.2004830.s023.pdf]

S1 Table. Data counts and accession numbers for sequence data used in this study.

Library name	Type	Read length	Insert size[1]	Number of read pairs (raw)	Number of read pairs (trimmed)	Number of bases (trimmed)	SRA accession
Aricci-PE	Short insert	100	250	190,627,875	131,683,730 (69.1%)	25,059,995,220 (65.7%)	ERR2135445
Aricci-MP1	Mate pair	51	3,000	137,584,511	124,345,538 (90.4%)	12,516,000,993 (89.2%)	ERR2135446
Aricci-MP2	Mate pair	51	3,000	71,764,090	67,858,702 (94.6%)	6,870,775,400 (93.9%)	ERR2135447
Aricci-RNA	RNA-Seq	150	250	125,096,118	94,033,091 (75.2%)	26,923,625,245 (71.7%)	ERR2135448
Aricci-PB	PacBio	3,618 (N50)	NA	NA	196,704	505,466,108	ERR2135450
Rmacru-PE	Short insert	150	500	191,998,065	173,358,788 (90.3%)	46,402,883,364 (80.6%)	ERR2135453
Rmacru-MP	Mate pair	150	2,000	91,593,889	67,854,215 (74.1%)	17,075,922,666 (62.1%)	ERR2135454, ERR2135455
Rmagna-PE	Short insert	150	550	162,142,774	61,225,674 (37.8%)	18,344,371,056 (37.7%)	ERR2135451
Rmagna-MP	Mate pair	150	2,000	15,885,056	11,443,087 (72.0%)	2,379,670,216 (49.9%)	ERR2135452

[1] Based on library preparation.
