# Supplementary material for: Comparative genomics of bdelloid rotifers: Insights from desiccating and nondesiccating species
Source: PLoS Biol. 2018 Apr 24;16(4):e2004830. doi: 10.1371/journal.pbio.2004830 (PMC5916493; doi:10.1371/journal.pbio.2004830)
Supplement: S2 Table — (PDF) [file pbio.2004830.s024.pdf]

S2 Table. MCScanX collinearity metrics within genomes.

**Species**		***A. ricciae***	***A. vaga***	***A. vaga* (collapsed)**	***R. macrura***	***R. magnacalcarata***
Genes[1]		49857	57431	35424	24594	29359
Assembly N50 (kb)		277	260	27	73	53
Singletons (% genes)		1613 (3.2%)	1246 (2.2%)	7583 (21.4%)	4704 (19.1%)	6029 (20.5%)
Dispersed duplicates (%)		9153 (18.4%)	15198 (26.5%)	23418 (66.1%)	16090 (65.5%)	19149 (65.2%)
Proximal duplicates (%)		588 (1.2%)	779 (1.4%)	252 (0.7%)	181 (0.7%)	265 (0.9%)
Tandem duplicates (%)		1139 (2.3%)	1206 (2.1%)	1173 (3.3%)	717 (2.9%)	920 (3.1%)
Segmental duplicates[2] (%)		37342 (74.9%)	38961 (67.9%)	2998 (8.5%)	2881 (11.7%)	2988 (10.2%)
	Of which form homologs blocks (%)	36595 (73.4%)	37061 (64.5%)	0	0	0
	Of which form ohnologous blocks (%)	17051 (34.2%)	15740 (27.4%)	2998 (8.5%)	2881 (100%)	2988 (100%)
Total # blocks						
	Homologous	475	905	0	0	0
	Ohnologous	903	923	184	175	187
Median block size (# genes)						
	Homologous	24	13	NA	NA	NA
	Ohnologous	11	9	5	11	12

[1]Curated gene sets for reference genomes were used in this analysis (see Table 1); [2]Total genes forming collinear blocks. Note that the a gene may contribute to multiple collinear blocks. Homologous blocks defined as those with an average pairwise Ks $\leq$ 0.5 (*A. ricciae*) and Ks $\leq$ 0.3 (*A. vaga*); ohnologous blocks defined as those with an average pairwise Ks > 0.5 (*A. ricciae*), Ks > 0.3 (*A. vaga*), and Ks > 0.4 for both *R. macrura* and *R. magnacalcarata*.
