# Supplementary material for: Comparative genomics of bdelloid rotifers: Insights from desiccating and nondesiccating species
Source: PLoS Biol. 2018 Apr 24;16(4):e2004830. doi: 10.1371/journal.pbio.2004830 (PMC5916493; doi:10.1371/journal.pbio.2004830)
Supplement: S3 Table — (PDF) [file pbio.2004830.s025.pdf]

S3 Table. Homologous and ohnologous K_A and K_S.

		Homologous blocks[1]		Ohnologous blocks			
**K_A**		***A. ricciae***	***A. vaga***	***A. ricciae***	***A. vaga***	***R. macrura***	***R. magnacalcarata***
N blocks		475	905	903	923	175	187
	Min	0.000	0.001	0.142	0.134	0.169	0.111
	Median	0.029	0.023	0.324	0.322	0.318	0.279
	Max	0.268	0.332	0.675	0.838	0.601	0.621
	Mean	0.036	0.032	0.332	0.333	0.333	0.292
	Stddev	0.026	0.034	0.066	0.082	0.086	0.083
	Stderr	0.001	0.001	0.002	0.003	0.007	0.006
**K_S**		***A. ricciae***	***A. vaga***	***A. ricciae***	***A. vaga***	***R. macrura***	***R. magnacalcarata***
	Min	0.000	0.003	0.695	0.369	0.537	0.467
	Median	0.132	0.041	1.273	0.616	0.705	0.698
	Max	0.403	0.297	1.809	0.807	0.947	0.898
	Mean	0.135	0.048	1.267	0.613	0.696	0.699
	Stddev	0.036	0.026	0.146	0.062	0.080	0.078

	Stderr	0.002	0.001	0.005	0.002	0.006	0.006
K_A/K_S		***A. ricciae***	***A. vaga***	***A. ricciae***	***A. vaga***	***R. macrura***	***R. magnacalcarata***
		0.267	0.667	0.262	0.543	0.478	0.418

[1]Homologous blocks defined as those with an average pairwise $K_S \leq 0.5$ (*A. ricciae*) and $K_S \leq 0.3$ (*A. vaga*); ohnologous blocks defined as those with an average pairwise Ks > 0.5 (*A. ricciae*), Ks > 0.3 (*A. vaga*).
