# Supplementary material for: Comparative genomics of bdelloid rotifers: Insights from desiccating and nondesiccating species
Source: PLoS Biol. 2018 Apr 24;16(4):e2004830. doi: 10.1371/journal.pbio.2004830 (PMC5916493; doi:10.1371/journal.pbio.2004830)
Supplement: S4 Table — (PDF) [file pbio.2004830.s026.pdf]

**S4 Table**. Species included in comparative analysis.

| Species name | Common name | Phylum | Genome span (Mb) | Scaffold N50 (kb) | Number of genes[1] | Accession |
|---|---|---|---|---|---|---|
| *Aplysia californica* | California sea hare | Mollusca | 927.3 | 91.7 kb | 21,312 | GCA_000002075.2 [1] |
| *Biomphalaria glabrata* | Freshwater snail | Mollusca | 916.4 | 48.1 kb | 28,551 | GCA_000457365.1 [2] |
| *Caenorhabditis elegans* | Roundworm | Nematoda | 100.3 | 17.5 kb | 20,222 | GCF_000002985.6 [3] |
| *Capitella teleta* | Annelid worm | Annelida | 333.3 | 188.4 kb | 32,175 | GCA_000328365.1 [4] |
| *Crassostrea gigas* | Pacific oyster | Mollusca | 557.7 | 401.7 kb | 26,101 | GCA_000297895.1 [5] |
| *Drosophila melanogaster* | Fruit fly | Arthropoda | 143.7 | 25,286.9 kb | 13,931 | GCA_000001215.4 [6] |
| *Helobdella robusta* | Leech | Annelida | 235.4 | 3,060.2 kb | 23,432 | GCA_000326865.1 [4] |
| *Intoshia linei* | NA | Orthonectida | 41.6 | 26.3 kb | 8,724 | GCA_001642005.1 [7] |
| *Lingula anatina* | Lampshell | Brachiopoda | 425.5 | 294.5 kb | 34,105 | GCA_001039355.1 [8] |
| *Lottia gigantea* | Owl limpet | Mollusca | 359.5 | 1,870.1 kb | 23,349 | GCA_000327385.1 [4] |

| | | | | | | |
|---|---|---|---|---|---|---|
| *Octopus bimaculoides* | Octopus | Mollusca | 2338.2 | 475.2 kb | 33,609 | GCA_001194135.1 [9] |
| *Ramazzottius varieornatus* | Water bear | Tardigrada | 55.8 | 474.0 kb | 19,521 | GCA_001949185.1 [10] |
| *Schistosoma haematobium* | Blood fluke | Platyhelminthes | 375.9 | 317.5 kb | 13,073 | GCA_000699445.1 [11] |

[1]Inferred from Ensembl Metazoa or the literature.

## References


1. Broad Institute [Internet]. [cited 2016]. Available: https://www.broadinstitute.org/aplysia/aplysia-genome-project
2. Adema CM, Hillier LW, Jones CS, Loker ES, Knight M, Minx P, et al. Whole genome analysis of a schistosomiasis-transmitting freshwater snail. Nat Commun. 2017;8: 15451.
3. C. elegans Sequencing Consortium. Genome sequence of the nematode C. elegans: a platform for investigating biology. Science. 1998;282: 2012–2018.
4. Simakov O, Marletaz F, Cho S-J, Edsinger-Gonzales E, Havlak P, Hellsten U, et al. Insights into bilaterian evolution from three spiralian genomes. Nature. 2013;493: 526–531.
5. Zhang G, Fang X, Guo X, Li L, Luo R, Xu F, et al. The oyster genome reveals stress adaptation and complexity of shell formation. Nature. 2012;490: 49–54.
6. Adams MD, Celniker SE, Holt RA, Evans CA, Gocayne JD, Amanatides PG, et al. The genome sequence of Drosophila melanogaster. Science. 2000;287: 2185–2195.
7. Mikhailov KV, Slyusarev GS, Nikitin MA, Logacheva MD, Penin AA, Aleoshin VV, et al. The Genome of Intoshia linei Affirms Orthonectids as Highly Simplified Spiralians. Curr Biol. 2016;26: 1768–1774.
8. Luo Y-J, Takeuchi T, Koyanagi R, Yamada L, Kanda M, Khalturina M, et al. The *Lingula* genome provides insights into brachiopod evolution and the origin of phosphate biomineralization. Nat Commun. 2015;6: 8301.
9. Albertin CB, Simakov O, Mitros T, Wang ZY, Pungor JR, Edsinger-Gonzales E, et al. The octopus genome and the evolution of cephalopod neural and morphological novelties. Nature. 2015;524: 220–224.
10. Hashimoto T, Horikawa DD, Saito Y, Kuwahara H, Kozuka-Hata H, Shin-I T, et al. Extremotolerant tardigrade genome and improved radiotolerance of human cultured cells by tardigrade-unique protein. Nat Commun. 2016;7: 12808.
11. Young ND, Jex AR, Li B, Liu S, Yang L, Xiong Z, et al. Whole-genome sequence of Schistosoma haematobium. Nat Genet. 2012;44: 221–225.

15. Young ND, Jex AR, Li B, Liu S, Yang L, Xiong Z, et al. Whole-genome sequence of Schistosoma haematobium. Nat Genet. 2012;44: 221–225.
