# Supplementary material for: Comparative genomics of bdelloid rotifers: Insights from desiccating and nondesiccating species
Source: PLoS Biol. 2018 Apr 24;16(4):e2004830. doi: 10.1371/journal.pbio.2004830 (PMC5916493; doi:10.1371/journal.pbio.2004830)
Supplement: S5 Table — (PDF) [file pbio.2004830.s027.pdf]

S5 Table. OrthoFinder clustering metrics.

	**Bdelloidea**	**Protostomia**
Number of genes in analysis (total)	161,241	519,293
Number of genes assigned to OGs	135,735 (84.2%)	440,684 (84.9%)
Number of unassigned genes	25,506 (15.8%)	78,609 (15.1%)
Number of OGs (total)	22,377	24,931
Number of species-specific OGs[1]	137	1,510
Number of genes in species-specific OGs	418 (0.3%)	9,342 (1.8%)
Mean OG size	6.1	17.7
Median OG size	6	7
G50[2] (assigned genes)	6	33
G50 (all genes)	6	27
O50[3] (assigned genes)	7,077	2,814
O50 (all genes)	9,204	4,148
Number of orthogroups with all species present	11,930 (53.3%)	1,894 (7.6%)
Number of single-copy orthogroups	104 (0.5%)	0

[1]An orthogroup containing only conspecific genes; [2]Cluster size at which 50% of genes are in an OG of that size or greater; [3]Fewest number of OGs required to reach G50.
