# Supplementary material for: Comparative genomics of bdelloid rotifers: Insights from desiccating and nondesiccating species
Source: PLoS Biol. 2018 Apr 24;16(4):e2004830. doi: 10.1371/journal.pbio.2004830 (PMC5916493; doi:10.1371/journal.pbio.2004830)
Supplement: S6 Table — (PDF) [file pbio.2004830.s028.pdf]

S6 Table. OrthoFinder clustering metrics per species.

BDELLOID CLUSTERING					
**Species**	**Proteins**	**Assigned (%)**	**OGs containing species (%)**	**Species-specific OGs**	**Proteins in species-specific OGs (%)**
A. ricciae	49,857	44,305 (88.9%)	19,816 (88.6%)	41	104 (0.2%)
A. vaga	57,431	46,230 (80.5%)	19,952 (89.2%)	65	196 (0.3%)
R. macrura	24,594	20,822 (84.7%)	15,764 (70.4%)	18	68 (0.3%)
R. magnacalcarata	29,359	24,378 (83%)	17,529 (78.3%)	13	50 (0.2%)
PROTOSTOMIA CLUSTERING					
**Species**	**Proteins**	**Assigned (%)**	**OGs containing species (%)**	**Species-specific OGs**	**Proteins in species-specific OGs (%)**
A. californica	27,591	25,169 (91.2%)	10,394 (41.7%)	50	219 (0.8%)
A. ricciae	49,857	47,562 (95.4%)	14,815 (59.4%)	51	121 (0.2%)
A. vaga	57,431	53,778 (93.6%)	15,106 (60.6%)	89	241 (0.4%)
B. glabrata	36,675	31,970 (87.2%)	10,594 (42.5%)	90	426 (1.2%)
C. elegans	28,137	16,602 (59%)	5,613 (22.5%)	217	1,876 (6.7%)
C. gigas	45,406	39,307 (86.6%)	10,115 (40.6%)	94	661 (1.5%)

C. teleta	31,978	25,781 (80.6%)	9,802 (39.3%)	86	757 (2.4%)
D. melanogaster	30,482	23,640 (77.6%)	6,286 (25.2%)	202	873 (2.9%)
H. robusta	23,426	17,408 (74.3%)	7,198 (28.9%)	50	591 (2.5%)
I. linei	8,724	6,293 (72.1%)	4,003 (16.1%)	58	347 (4%)
L. anatina	43,670	39,507 (90.5%)	9,831 (39.4%)	179	958 (2.2%)
L. gigantea	23,822	20,400 (85.6%)	10,003 (40.1%)	54	1,003 (4.2%)
O. bimaculoides	23,994	21,986 (91.6%)	8,495 (34.1%)	78	280 (1.2%)
R. macrura	24,594	23,062 (93.8%)	12,235 (49.1%)	16	51 (0.2%)
R. magnacalcarata	29,359	27,274 (92.9%)	13,401 (53.8%)	14	50 (0.2%)
R. varieornatus	23,007	12,976 (56.4%)	5,976 (24%)	132	630 (2.7%)
S. haematobium	11,140	7,969 (71.5%)	5,078 (20.4%)	50	258 (2.3%)
