# Supplementary material for: Comparative genomics of bdelloid rotifers: Insights from desiccating and nondesiccating species
Source: PLoS Biol. 2018 Apr 24;16(4):e2004830. doi: 10.1371/journal.pbio.2004830 (PMC5916493; doi:10.1371/journal.pbio.2004830)
Supplement: S1 Text — (PDF) [file pbio.2004830.s029.pdf]

## S1 Text. Signals of duplication and collapse in the *A. ricciae* sequence data

A number of observations support the conclusion that only a minority of homologous gene copies are collapsed in the *A. ricciae* reference genome. First, the majority of *A. ricciae* genes (~73%) have a homologous partner (defined by $K_S \leq 0.5$) that is separately assembled elsewhere in the reference (**Fig 2A**; **S2 Table**), and almost all (~99%) such genes show the putative twofold coverage value of 150x (**S10A Fig**). Thus, twofold coverage cannot be solely the result of collapse between homologous gene copies. Furthermore, scrutiny of SNP densities across individual genes reveals a high degree of heterogeneity in SNP density: the majority of genes have zero or very few detected SNPs (47,803, ~82%), and only relatively few genes (5,544, ~9%) show ≥ 1% SNP density (**S10B Fig**). If homologous collapse were more widespread or complete (as inferred in *Rotaria*), we would expect a higher number of SNPs across all genes, particularly if homologous divergence were relatively high. Taken together, these results indicate unequal stoichiometry of DNA molecules in the *A. ricciae* sequence data (generated from multiple clonal individuals and cell types), whose signal is further complicated by partial assembly collapse. Under this hypothesis, the majority of the genome is represented in the data at twofold stoichiometry (150x coverage), and a lesser proportion at onefold (75x). The SNPs at 50% frequency and 150x coverage are then due to assembly collapse involving onefold regions. Given that the embryos of both *A. ricciae* and *A. vaga* appear to have 12 chromosomes [1,2], this pattern is probably not due to an increase in germline ploidy. One explanation could be endopolyploidy [3], whereby some (but not all) chromosomes are maintained in multiple copies in somatic tissues of *A. ricciae* adults. Alternatively, these signals may represent an additional stratum of duplication in the *A. ricciae* lineage that was not evident from karyotype visualisation, and whose evolutionary significance is undetermined. Further investigations of the *A. ricciae* genome data are required to test these hypotheses.

## References


1. Mark Welch JL, Meselson M. Karyotypes of bdelloid rotifers from three families. Rotifera VIII: A Comparative Approach. Springer, Dordrecht; 1998. pp. 403–407.

2. Pouchkina-Stantcheva NN, McGee BM, Boschetti C, Tolleter D, Chakrabortee S, Popova AV, et al. Functional divergence of former alleles in an ancient asexual invertebrate. Science. 2007;318: 268–271.

3. Jones PA, Gilbert JJ. Polymorphism and polyploidy in the rotifer Asplanchna sieboldi: Relative nuclear DNA contents in tissues of saccate and campanulate females. J Exp Zool. Wiley Subscription Services, Inc., A Wiley Company; 1977;201: 163–168.
