# Supplementary material for: Comparative genomics of bdelloid rotifers: Insights from desiccating and nondesiccating species
Source: PLoS Biol. 2018 Apr 24;16(4):e2004830. doi: 10.1371/journal.pbio.2004830 (PMC5916493; doi:10.1371/journal.pbio.2004830)
Supplement: S2 Text — (PDF) [file pbio.2004830.s030.pdf]

S2 Text. Note on bdelloid genome sizes

Mark Welch & Meselson (2003) [1] conducted fluorometric DNA quantitation in oocytes to assess genome size in a number of bdelloid species, including *A. vaga* and *A. ricciae* (then called "*Adineta* sp.1" [2]). The total oocyte DNA content was estimated to be ca. 0.49 pg (479 Mb) for *A. ricciae* and ca. 0.74 pg (724 Mb) for *A. vaga* (Table 3 of [1]). However, when these values were imported to the Animal Genome Size Database [3] (www.genomesize.com), they were accidentally switched (and then divided by two to derive "haploid" C-values, which is arguably inapplicable to the asexual bdelloids). Between December 2003 and January 2018, this online resource erroneously reported a C-value of 0.36 pg for *A. ricciae* (352 Mb), and of 0.25 pg for *A. vaga* (244 Mb). This last value was cited by Flot et al. [4] when validating the assembly of *A. vaga*; by coincidence, the database entry appeared to confirm their size estimate of ca. 250 Mb (based on assembly span and coverage analysis). The transposition error in the online database has been corrected as of February 2018 (T. Ryan Gregory, pers comm.) and it is now clear that the fluorometric measurements of absolute genome size [1] differ substantially from assembly size, both as estimated previously [4] and in this work. Even in relative terms, the fluorometric data [1] suggest that *A. ricciae* has a markedly smaller genome than *A. vaga* (~66%), which is not reflected in the relative assembly sizes. The reason for these substantial discrepancies between fluorometric and bioinformatic results is unclear (David Mark Welch, pers comm.). Owing to these uncertainties, cytological measures of genome size were not used to validate the *A. ricciae* assembly or to interpret other patterns in these data.

References

1. Mark Welch DB, Meselson M. Oocyte nuclear DNA content and GC proportion in rotifers of the anciently asexual Class Bdelloidea. Biol J Linn Soc Lond. Blackwell Science Ltd; 2003;79: 85–91.

2. Segers H, Shiel R. Tale of a sleeping beauty: a new and easily cultured model organism for experimental studies on bdelloid rotifers. Rotifera X. Springer; 2005; 141–145.

3. Gregory TR. Animal Genome Size Database [Internet]. [cited 2018]. Available: http://www.genomesize.com

4. Flot J-F, Hespeels B, Li X, Noel B, Arkhipova I, Danchin EGJ, et al. Genomic evidence for ameiotic evolution in the bdelloid rotifer Adineta vaga. Nature. 2013;500: 453–457.
