# Supplementary material for: Comparative genomics of bdelloid rotifers: Insights from desiccating and nondesiccating species
Source: PLoS Biol. 2018 Apr 24;16(4):e2004830. doi: 10.1371/journal.pbio.2004830 (PMC5916493; doi:10.1371/journal.pbio.2004830)
Supplement: S3 Text — (PDF) [file pbio.2004830.s031.pdf]

S3 Text. Test of desiccation tolerance for *Rotaria* species

Desiccation tolerance was assessed for *R. macrura* using a version of a protocol described elsewhere [1]. Individuals were taken from a laboratory culture derived from wild-caught founders, sourced and maintained as described above. As a positive control, desiccation tolerance also was measured for an undescribed species of *Macrotrachela*, collected from terrestrial moss at Silwood Park, Ascot, UK (51° 24' 29.1" N, 0° 38' 42.5'" W) and maintained as a single clone in culture since 22nd June 2015. The source cultures for both species were fed and reared under the same conditions. Approximately four individuals were moved by pipette into each of 14 35mm Petri dishes, and counted (*n* = 28 animals for *R. macrura*; *n* = 27 animals for *Macrotrachela* sp.). To these was added 2.5 mL sterile distilled water, and three 5x5 mm squares of autoclaved Whatman filter paper, to provide a substrate for desiccation. Dishes were placed into a controlled environmental chamber (Memmert ICH 100), with dishes for each species interspersed. Their lids were removed and the relative humidity (RH) was reduced in a linear progression from 80% to 40% over 16 hours, then held at 40%. After 14 days, RH was increased from 40% to 60% over 90 minutes, and the dishes were rehydrated individually with 2.5 mL sterile distilled water. The temperature was maintained at 18°C throughout. Animals were re-counted after 3.5 hours, and again after 21 hours. They were classed as active (if feeding or locomoting), dead (if immobile with degradation of organ structure), or contracted (if immobile but without clear evidence of internal degradation). Survival was calculated for each species as the number of animals active at the 21 hour count, divided by the total number of animals prior to desiccation. The survival of *Macrotrachela* sp. was 48.1%, but there was zero survival for *R. macrura*; at 21 hours after rehydration, all desiccated individuals of *R. macrura* were clearly dead. It cannot be excluded that *R. macrura* survives desiccation rarely or under specialised conditions in nature, but no desiccation tolerance was detected here, even though this protocol has previously been successful for many other species, including *Rotaria neptunoida*, which also lives in typically permanent bodies of water [1]. Individuals of *R. magnacalcarata* were previously found not to survive desiccation in plastic dishes with a filter paper substrate [2]. In nature, however, this species lives epibiotically in groups on the underside of the waterlouse *Asellus aquaticus* [3]. We investigated whether *R. magnacalcarata* might tolerate desiccation better when associated with its host. We took five *Asellus* individuals from the location described by [2]. Each was carrying at least five rotifers belonging to *R. magnacalcarata*, along with some individuals of *R. socialis*, although a precise count was not possible in situ. The lice were placed in a single 90mm Petri dish along with water from their original location. As a positive control, several hundred lab-grown individuals of *A. ricciae* were added, to be desiccated at the same time. The dish was desiccated in an environmental chamber, as described above. RH was reduced from 80% to 40% over 24 hours, then maintained at

40% RH for 48 hours, then increased to 60% over 90 minutes, and the dish was rehydrated with 10mL of distilled water. The temperature was maintained at 10°C throughout, approximating the temperature of the original habitat. No surviving waterlice or epibiotic rotifers were found either at 3 hours or 24 hours after rehydration, though the co-desiccated population of *A. ricciae* recovered as expected. After rehydration, the dead *Rotaria* were found to be floating separately from their hosts, though it was unclear whether detachment occurred actively before desiccation was complete or passively during rehydration. Embryos were present in some of the corpses, but showed no sign of life, in contrast to the survival of embryos after desiccation in *R. neptunoida*. There is no evidence that *R. magnacalcarata* (or *R. socialis*) can tolerate desiccation even when attached to their hosts, though it is very difficult to exclude the possibility that some low level of desiccation tolerance occurs in nature under specific conditions.

References

1. Ricci C. Anhydrobiotic capabilities of bdelloid rotifers. Hydrobiologia. Kluwer Academic Publishers; 1998;387-388: 321–326.

2. Eyres I, Boschetti C, Crisp A, Smith TP, Fontaneto D, Tunnacliffe A, et al. Horizontal gene transfer in bdelloid rotifers is ancient, ongoing and more frequent in species from desiccating habitats. BMC Biol. 2015;13: 90.

3. Fontaneto D, Ambrosini R. Spatial niche partitioning in epibiont rotifers on the waterlouse *Asellus aquaticus*. Limnol Oceanogr. 2010;55: 1327–1337.
